# Research Priorities of Applying Low-Cost PM_2.5_ Sensors in Southeast Asian Countries

**DOI:** 10.3390/ijerph19031522

**Published:** 2022-01-28

**Authors:** Shih-Chun Candice Lung, To Thi Hien, Maria Obiminda L. Cambaliza, Ohnmar May Tin Hlaing, Nguyen Thi Kim Oanh, Mohd Talib Latif, Puji Lestari, Abdus Salam, Shih-Yu Lee, Wen-Cheng Vincent Wang, Ming-Chien Mark Tsou, Tran Cong-Thanh, Melliza Templonuevo Cruz, Kraichat Tantrakarnapa, Murnira Othman, Shatabdi Roy, Tran Ngoc Dang, Dwi Agustian

**Affiliations:** 1 Research Center for Environmental Changes, Academia Sinica, Taipei 115, Taiwan; shihyu@gate.sinica.edu.tw (S.-Y.L.); phdzen@gate.sinica.edu.tw (W.-C.V.W.); marktsou09@gate.sinica.edu.tw (M.-C.M.T.); 2 Department of Atmospheric Sciences, National Taiwan University, Taipei 106, Taiwan; 3 Faculty of Environment, University of Science, Ho Chi Minh City 700000, Vietnam; tohien@hcmus.edu.vn (T.T.H.); tcthanh@hcmus.edu.vn (T.C.-T.); 4 Vietnam National University, Ho Chi Minh City 700000, Vietnam; 5 Department of Physics, Ateneo de Manila University, Quezon City 1108, Philippines; mcambaliza@ateneo.edu; 6 Air Quality Dynamics Laboratory, Manila Observatory, Quezon City 1108, Philippines; liz@observatory.ph; 7 Environmental Quality Management Co., Ltd., Yangon 11072, Myanmar; ohnmarmay@gmail.com; 8 Environmental Engineering and Management, SERD, Asian Institute of Technology, Pathumthani 12120, Thailand; kimoanh@ait.asia; 9 Department of Earth Sciences and Environment, Universiti Kebangsaan Malaysia, Bangi 43600, Malaysia; talib@ukm.edu.my; 10 Faculty of Civil and Environmental Engineering, Bandung Institute of Technology, Bandung 40132, Indonesia; pujilest@indo.net.id; 11 Department of Chemistry, Faculty of Science, University of Dhaka, Dhaka 1000, Bangladesh; asalam@du.ac.bd (A.S.); shatabdi@du.ac.bd (S.R.); 12 College of Public Health, National Taiwan University, Taipei 100, Taiwan; 13 Department of Social and Environmental Medicine, Faculty of Tropical Medicine, Mahidol University, Bangkok 10400, Thailand; kraichat.tan@mahidol.ac.th; 14 Institute for Environment and Development (Lestari), Universiti Kebangsaan Malaysia, Bangi 43600, Malaysia; murnira@ukm.edu.my; 15 Department of Environmental Health, Faculty of Public Health, University of Medicine and Pharmacy at Ho Chi Minh City, Ho Chi Minh 700000, Vietnam; ngocdangytcc@gmail.com; 16 Department of Public Health, Faculty of Medicine, Universitas Padjadjaran, Bandung 40171, Indonesia; dwi.agustian@unpad.ac.id

**Keywords:** low-cost PM_2.5_ sensors, Hi-ASAP, transdisciplinary research, air quality and health, PM_2.5_ regional transport, Asian PM sources, exposure and health relationships, co-benefit of climate and health

## Abstract

The low-cost and easy-to-use nature of rapidly developed PM_2.5_ sensors provide an opportunity to bring breakthroughs in PM_2.5_ research to resource-limited countries in Southeast Asia (SEA). This review provides an evaluation of the currently available literature and identifies research priorities in applying low-cost sensors (LCS) in PM_2.5_ environmental and health research in SEA. The research priority is an outcome of a series of participatory workshops under the umbrella of the International Global Atmospheric Chemistry Project–Monsoon Asia and Oceania Networking Group (IGAC–MANGO). A literature review and research prioritization are conducted with a transdisciplinary perspective of providing useful scientific evidence in assisting authorities in formulating targeted strategies to reduce severe PM_2.5_ pollution and health risks in this region. The PM_2.5_ research gaps that could be filled by LCS application are identified in five categories: source evaluation, especially for the distinctive sources in the SEA countries; hot spot investigation; peak exposure assessment; exposure–health evaluation on acute health impacts; and short-term standards. The affordability of LCS, methodology transferability, international collaboration, and stakeholder engagement are keys to success in such transdisciplinary PM_2.5_ research. Unique contributions to the international science community and challenges with LCS application in PM_2.5_ research in SEA are also discussed.

## 1. Introduction

The World Health Organization (WHO) recently announced the revised recommendations for air-quality guidelines to urge authorities worldwide to effectively reduce air pollution for the protection of human health [1]. Among air pollutants, particulate matter with an aerodynamic diameter less than or equal to 2.5 microns (PM_2.5_) is regarded as a major health threat, especially in Asia. It has been estimated that 2.2 million of the world’s 7 million premature deaths each year that are attributable to household (indoor) and ambient (outdoor) air pollution occurred in Asia [2], including deaths from both acute and chronic health impacts. Chronic impacts such as chronic obstructive pulmonary disease and lung cancer are associated with long-term exposures, whereas the acute impacts such as asthmatic attacks and the irregularity of heart conditions (which may lead to stroke or other heart diseases) are directly associated with peak exposures, which have been difficult to measure up until now. Rapidly developed sensor technology has shed light on tackling this challenge.

The quickly-growing body of publications in applying low-cost sensors (LCS) in PM research has shown great potential in filling research gaps in current PM_2.5_ environmental and health studies in Asia [3,4,5]. The LCS referred to in this review paper are sensors with powerful single board computer platforms (e.g., Arduino, Raspberry Pi, and the STM32 family), which are much less expensive than previous platforms [3]. The low consumption of the battery power in such computers makes LCS much smaller and lighter than traditional instruments. In 2015, a review indicated that LCS with good precision could be used by citizens as an indicative tool to show the relative pollution levels among different conditions; nevertheless, the drawback of failing to provide accurate measurements limited their application in environmental research at that time [4]. Fortunately, the rapid development of sensors and calibration techniques/methods has greatly enhanced the data quality of LCS [6]. In 2020, most of the 50 LCS models for PM evaluated in a review could accurately monitor PM changes in the environment and exhibit good performance, with accuracy reaching R^2^ up to 0.99 in some conditions after calibration [3]. For example, in comparison with reference instruments, Plantower PMS 7003 sensors had an R^2^ of 0.96 and precision within 16% in the PM_2.5_ concentration range of 16–75 μg/m^3^; and Novasense SDS011 sensors had an R^2^ of 0.90 and precision within 12% in the PM_2.5_ concentration range of 0–120 μg/m^3^ [3]. This evidence shows that LCS devices are ready for use in environmental research. The inexpensive and easy-to-use nature of LCS enables scientists in resource-limited countries in Southeast Asia (SEA) to carry out studies that have previously been conducted with expensive instruments. In addition, the fast-response sensors can detect PM_2.5_ levels in sub-minute time resolutions. This provides a great opportunity for SEA scientists to conduct PM_2.5_ environmental and health studies for populations exposed to certain distinctive Asian sources with peak exposures (e.g., [7]). 

To seize this opportunity, an initiative called the Health Investigation and Air Sensing for Asian Pollution (Hi-ASAP) was developed under the umbrella of the Monsoon Asia and Oceania Networking Group (MANGO) of the International Global Atmospheric Chemistry Project (IGAC). IGAC was established in 1990 with the mission of facilitating atmospheric chemistry research towards a sustainable world (https://igacproject.org/igac-welcome, accessed on 14 October 2021) and is currently operated under Future Earth (https://futureearth.org/, accessed on 14 October 2021). In 2015, IGAC–MANGO was formed to strengthen the capability of Asian scientists in conducting atmospheric chemistry research. In line with the transdisciplinary research that Future Earth promotes, the main goal of the Hi-ASAP is to provide scientific evidence to support effective policy actions to reduce air pollution levels, particularly PM_2.5_, in this region by applying newly developed LCS. 

The region of SEA and South Asia had the highest annual population-weighted PM_2.5_ concentrations in 2019 [1], which highlights the importance and urgency of PM_2.5_ reduction in this region. The announcement of PM_2.5_ levels used to be the privilege of the authorities in the SEA region, which set up monitoring stations in limited locations with expensive instruments. The small and easy-to-carry LCS empowers scientists to identify sources in locations without official monitoring stations and quantify the impacts of sources on ambient air quality and residents’ exposures (e.g., [8,9]). The waterproof housing of the LCS further reduces the cost of PM_2.5_ monitoring, since air-conditioned rooms are no longer required as they were for traditional instruments. Multiple deployments of the affordable and fast-response LCS provides much finer temporal and spatial PM_2.5_ distributions than ever before. Thus, LCS may contribute to source identification, hot spot evaluation, and peak exposure assessment covering much broader areas and populations. These extra findings can assist authorities in prioritizing/refining source control strategies, especially in densely populated SEA countries where emission sources are scattered within or around peoples’ living environments (e.g., [9]). Additionally, behavioral change tactics could be formulated by the authorities based on the identified peak exposure microenvironments/activities for high-exposure and susceptible subpopulations to reduce their exposures and health risks. Furthermore, the data of LCS could be transmitted in real-time and shared to the general public much easier via social media, pressuring authorities for better enforcement and industry for better air pollution control. These are all potential policy-relevant contributions of applying LCS in PM_2.5_ research in SEA.

The COVID-19 pandemic led to dramatic improvements in air quality worldwide in 2020 for a brief period [10]. The blue sky that had been masked for over for decades, especially in developing countries, was unveiled through many photos on social media; those eye-catching demonstrations have raised people’s expectations for better air quality. The United Nations Development Programme further emphasized that “COVID-19 is forcing us to revisit our values and design a new area of development that truly balances economic, social and environmental progress as envisioned by the 2030 Agenda and the sustainable development goals (SDGs)” (https://feature.undp.org/covid-19-and-the-sdgs/, accessed on 16 October 2021). The authorities need to have a different perspective in the post-COVID-19 era in managing air pollution to protect public health and achieve SDGs [11]. Minimizing fossil fuel sources could contribute to both health risk reduction and climate mitigation. This highlights the urgency of providing solid scientific evidence with new technology, such as LCS, to support effective policy actions to phase down fossil fuel sources and reduce PM_2.5_ for public health and climate protection, especially in SEA. 

This review evaluates the current literature focusing on the application of low-cost PM_2.5_ sensors (LCPMS) in environmental and health research in the SEA region and identifies priority research areas with the specific aim of providing scientific evidence to assist policymakers in prioritizing source controls, identifying hot spots, and formulating behavioral change tactics for PM_2.5_ health risk reduction. The priority research areas were identified through a series of participatory workshops under the support of IGAC–MANGO and other international collaborators, including Integrated Research on Disaster Risk, the International Centre of Excellence in Taipei (IRDR ICoE-Taipei), the International Science Council Regional Office for Asia and the Pacific (ISC ROAP), the Regional Office of Future Earth in Asia, Future Earth Taipei, and Academia Sinica. This paper presents the review framework and process in the Materials and Methods section. The literature reviews under different topics and the identified research priorities are shown in the Results section. The unique angles, contributions to the international research arena, and potential challenges by conducting PM_2.5_ research in the SEA region using LCPMS are shared in the Discussion section. 

## 2. Materials and Methods 

**Figure 1:**
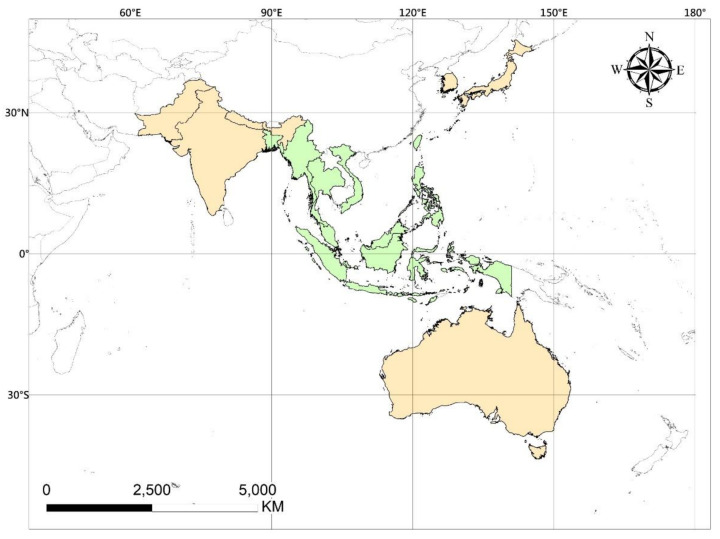
The geographic distribution of the participating research groups in the 2019 workshop by their respective countries (colored); countries in green are the eight countries included in this paper.

**Figure 2:**
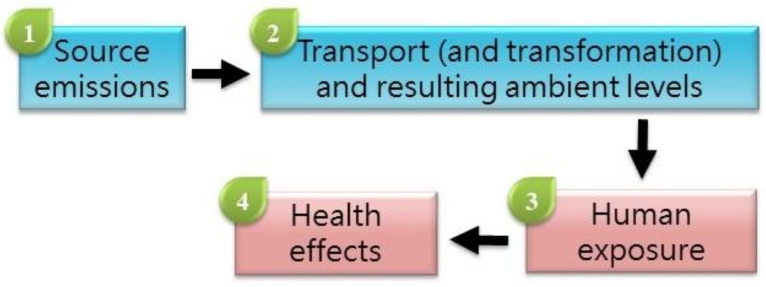
Review framework based on the progression of emission sources to health impacts. Numbers in the figure are research focuses reviewed in this paper. Blue indicates environment-related and red indicates human-related focuses.

Hi-ASAP consists of atmospheric chemistry and public health scientists in the Asian region interested in taking on transdisciplinary perspectives in conducting air pollution and health research with LCPMS. The development of the research agenda was first discussed in the Hi-ASAP planning workshop held in Academia Sinica, Taipei, Taiwan, on 17–19 May 2019, in the presence of researchers from 14 countries in the Asian and Pacific regions (Figure 1); the participants are listed in Appendix A. The rich inputs and fruitful discussion in this workshop were synthesized as the Hi-ASAP Science and Implementation Plan endorsed by the Regional Advisory Committee of the Regional Office of Future Earth in Asia in October 2019; thus, Hi-ASAP is an activity of Future Earth Asia. Afterward, this Science and Implementation Plan was discussed and revised in the online Hi-ASAP workshops in October 2020 and October 2021. The current literature on PM_2.5_ environmental and health research using LCPMS was searched and evaluated to revise the research priorities. The following paragraphs briefly describe the Hi-ASAP research framework based on the progression of PM_2.5_ emission sources to health impacts (Figure 2), which is also used in this paper as the review framework. 

To provide useful scientific evidence for policymakers to effectively reduce PM_2.5_ levels and the associated health risks, scientists must evaluate important and controllable factors in the progress of emission sources to health impacts. The evaluated controllable factors can be translated into formulating intervention strategies that can be embedded in various environmental and health policies aiming to reduce PM_2.5_ levels and health risks. With a transdisciplinary perspective in mind, assessing important controllable factors is key to the successful translation of scientific findings into policy recommendations. Four research focuses, each with controllable factors, along this progression are identified as indicated in Figure 2. 

Briefly, after PM_2.5_ and co-pollutants are emitted from sources, primary PM_2.5_ transported by air mass movement and secondary PM_2.5_ formed via photochemical transformation are mixed as ambient PM_2.5_. Thus, the evaluation of source characteristics is the first research focus (Figure 2). Since LCPMS cannot differentiate primary and secondary PM_2.5_, the second research focus includes transport/transformation and levels in ambient air. The features of fast-response and small LCPMS combined with a solar power supply and waterproof housing can contribute to providing PM_2.5_ levels with fine resolutions in near-source evaluation and ambient air for the first two research focuses (e.g., [8,9]). The controllable factors evaluated in these two research focuses are the sources themselves and the hot spots identified. In the Web of Science search conducted on 24 October 2021, there were 109 publications with the keywords “low-cost sensor”, “PM_2.5_”, and “source”; more than half (62) were conducted in the US. Among Asian countries, China, Taiwan, India, and South Korea had 15, 11, 7, and 7 publications, respectively. Japan, Indonesia, Malaysia, Vietnam, and Singapore all had only one publication per country. In addition, there were 140 publications with the keywords “low-cost sensor”, “PM_2.5_”, and “ambient air”, with 82 from the US. China, Taiwan, and India had 21, 13, and 11 publications, respectively, whereas South Korea and Japan had 4 and 2 publications, respectively. Indonesia, Malaysia, and Singapore all had only one publication each. This quickly shows the general landscape of the current LCPMS publications in the first two research focuses. 

After people are exposed to PM_2.5_, certain acute or chronic health effects may develop. Thus, human exposure and the exposure–health relationships are the third and fourth research focuses, respectively (Figure 2). Fast-response LCS can contribute to identifying peak PM_2.5_ exposures and the relevant exposure microenvironments and exposure activities (e.g., [7,12]); these are controllable factors evaluated in these two research focuses. In addition, the advantages of being small, lightweight, and easy to carry without noise and vibration make LCS good research tools in panel-type epidemiological studies for recruiting subjects in assessing actual exposure and the exposure–health relationships of ordinary citizens and high-exposure or high-susceptible subpopulations (e.g., [13,14]). In the search with the keywords “low-cost sensor”, “PM_2.5_”, and “exposure”, there were 226 publications, with 119 from the US. China, Taiwan, India, and South Korea had 31, 14, 11, and 8 publications, respectively, whereas Japan, Indonesia, Vietnam, and Singapore had 2, 3, 2, and 1 publications, respectively. For the combination of the keywords “low-cost sensor”, “PM_2.5_”, and “exposure and health”, there were 125 publications, with 65 from the US. China, Taiwan, India, and South Korea had 18, 8, 6, and 7 publications, respectively, whereas Japan, Indonesia, Vietnam, and Singapore had only 1, 2, 2, and 1 publications, respectively.

The literature review presented in this paper focuses on SEA countries; therefore, publications from the US, China, India, South Korea, and Japan are not included unless noted otherwise. Taiwan is included for two reasons: (1) it serves as a subtropical country for comparison, and (2) the Taiwan research group acts as the LCPMS hub in the Hi-ASAP for transferring technology and methodologies to SEA research groups. The importance of international collaboration for PM_2.5_ research with LCPMS will be discussed in the Discussion section. As of November 2021, some researchers in the 2019 Hi-ASAP workshop were no longer able to join the team. Thus, the following literature review focuses on only eight countries: Bangladesh, Indonesia, Malaysia, Myanmar, Philippines, Taiwan, Thailand, and Vietnam (green areas shown in Figure 1). This includes all SEA countries in the aforementioned Web of Science search, except Singapore, which is not included in the summary in Table 1 and Table 2, but the relevant publications are included under different categories of sub-sections in this review.

Table 1a summarizes the characteristics of these eight countries with population densities (calculated based on population estimates in 2019 and total areas of these countries) ranging from 79.9 person/km^2^ in Myanmar to 1132.3 person/km^2^ in Bangladesh. The gross domestic product (GDP) per capita and employment percentage in industry clearly show that these countries are at different stages of industrial development. The population and population densities of their capital cities in 2019, as well as PM_2.5_ levels from the ground-level monitoring stations of 2019 and 2020 in these cities, are also presented in Table 1b. The impacts of COVID-19 on PM_2.5_ reductions are clearly demonstrated. Nevertheless, the order of PM_2.5_ levels in these cities was the same for both years, which was not related to the order of their population or population densities. Taiwan had the highest GDP per capita and highest employment percentage in industry with the lowest PM_2.5_ in 2019 and 2020 in the capital city. Typically, PM_2.5_ levels are reversely related to the stage of industrial development. At a higher stage, PM_2.5_ levels are usually lower due to better environmental policies and enforcement. However, PM_2.5_ levels in the capital cities of these countries did not exactly follow the opposite order of GDP per capita or percentage of employment in industry. For example, Manila in the Philippines had the second-lowest PM_2.5_ among eight capital cities in both 2019 and 2020, whereas the GDP per capita of the Philippines ranked fifth (from high to low). In addition, with a similar percentage of employment in industry (27%), Hanoi in Vietnam had twice the PM_2.5_ levels of Kuala Lumpur in Malaysia. Moreover, Jakarta, Indonesia, had more than twice the PM_2.5_ levels of Bangkok, Thailand, despite both countries having similar employment percentages in industry. This discrepancy may be due to differences in regulations in industry zoning and source control surrounding the capital cities, in addition to geographical and meteorological factors. This again highlights the importance of environmental policies and enforcement on PM_2.5_ levels, regardless of the stage of industrial development. In short, this table demonstrates that the eight countries under review cover a wide range of development and PM_2.5_ levels. In fact, among 85 capital cities worldwide in 2019, Dhaka, Bangladesh had the second-highest PM_2.5_ levels (83.3 μg/m^3^), and Taipei, Taiwan, ranked 56th (13.9 μg/m^3^), with most Asian capital cities in between. Only Delhi, India (ranked 1st) and Tokyo, Japan (ranked 63th) were outside the range [28].

## 3. Results

According to the review framework, the results of the literature review are presented in the following order: source evaluation (Section 3.1), ambient monitoring and transport (Section 3.2), exposure assessment (Section 3.3), and exposure–health evaluation (Section 3.4). Publications in international journals are the review targets. However, governmental documents, newsletters from international organizations, conference presentations, or papers in local journals were mentioned briefly, in cases where no publications in international journals were found in certain categories or countries, to demonstrate the capability of local scientists in applying LCPMS. Some publications identified by the aforementioned keyword search in SEA may not have been included, since an in-depth evaluation found that certain keywords were briefly mentioned in those papers without investigation. In cases where no publications in SEA were found, publications in Asia were presented, serving as exemplars. Based on the literature review and discussion in the Hi-ASAP workshops in 2019–2021, the research gaps that can be filled by using LCPMS in SEA are presented in Section 3.5.

Here, a particular kind of LCPMS, AS-LUNG (short for Academia Sinica-LUNG), is described briefly since it is mentioned repeatedly in the following sections. AS-LUNG was integrated by the Taiwan research group at Academia Sinica and has been used by the Hi-ASAP team in the past few years. AS-LUNG uses a PMS3003 sensor (Plantower, Beijing, China) to assess PM_2.5_. Three different types were integrated for outdoor, indoor, and portable applications, namely AS-LUNG-O, AS-LUNG-I, and AS-LUNG-P, respectively [7,9]. Each AS-LUNG set was calibrated in the laboratory against research-grade instruments (GRIMM and EDM-180 from GRIMM Aerosol Technik Ainring GmbH & Co., Ainring, Germany) [29]. Compared to EDM-180, AS-LUNG sets had a high R^2^ of 0.999, less than 6.0% variability in the slopes, and a root mean square error (RMSE) of 1.18 μg/m^3^ for 0.1–200 μg/m^3^ of PM_2.5_ with segmented regressions [29]. Sensor readings were all corrected by regression equations accordingly to obtain good-quality data suitable for PM_2.5_ research.

### 3.1. Publications on Source Evaluation in SEA Using LCPMS

Section 3.1 is focused on reviewing articles evaluating distinctive sources in the SEA region that contribute significantly to ambient PM_2.5_ levels or personal PM_2.5_ exposures but have not been thoroughly investigated. Certain sources are culture-specific, whereas certain sources are old-fashioned practices that are seldom found in Western countries. In either case, these locally important sources are understudied in the current mainstream PM_2.5_ research community. Specifically, the review targets the sources of primary importance for the region, namely biomass and agriculture open burning, traffic emissions of locally made vehicles, Asian-style cooking and street cooking, incense burning, open waste burning, and fuel combustion for brick manufacturing. The publications that assessed these sources with traditional or state-of-the-art instruments are used to demonstrate the importance of these sources. Then, publications that assessed these sources with LCPMS are reviewed. The potential advantages of applying LCPMS are elaborated.

#### 3.1.1. Biomass and Agriculture Burning

Biomass and agricultural burning have been identified as major PM sources in Asian regions. Particularly during dry seasons, biomass and agricultural burning significantly impact the air quality in SEA, with high PM_2.5_ concentrations usually affected by regional wind directions, high temperatures, low rainfall, and phenomena such as the El Niño–Southern Oscillation (ENSO) [30,31]. Field campaigns for biomass and agriculture burning using LCPMS in Asia have been conducted, indicating high PM_2.5_ concentrations in Hanoi [32,33], the northern region of Thailand [34], and New Delhi [35] during biomass burning episodes; sometimes the PM_2.5_ levels exceeded 100 µg/m^3^. The PM_2.5_ concentrations were influenced by the number of hot spots and wind direction toward the sampling stations. The significant impacts of biomass burning on regional air quality in SEA were also confirmed by modeling works. For example, a regional model showed transport paths from Sumatra and on route to Kuala Lumpur with large amounts of PM [36].

PM_2.5_ concentrations using LCPMS in several parts of Asia affected by biomass burning episodes were similar to those using the research-grade instruments operated by government agencies. For example, in Malaysia, the highest PM concentration recorded during biomass-burning episodes of the southwest monsoon season using LCPMS was the same as that recorded using continuous monitoring instruments by the Malaysian Department of Environment [37]. In addition, the PM_2.5_ concentrations in Palangkaraya in Kalimantan during the biomass-burning episodes in 2019 were 36 to 384 µg/m^3^, obtained by the Ministry of Environment and Forestry in Indonesia. The PM_2.5_ concentrations in the northern areas of SEA during biomass burning were between 80.8 and 93.1 µg/m^3^, as assessed by the Seven South-East Asian Studies (7-SEAS) [38]. All these results show that the PM_2.5_ concentrations using LCPMS were within the range of PM_2.5_ obtained with the research-grade instruments.

#### 3.1.2. Transportation

The transportation sector is one of the major contributors to the atmospheric PM_2.5_ burden in SEA cities. In 2015, the emissions of PM_2.5_ in SEA was estimated to be 2.5 Tg/yr., with about a 16% contribution from black carbon (BC) [39], one of the important PM_2.5_ components with health concerns [40]. Although BC emissions from road transport seemed to be on a declining trend in the late 2000s, presumably because of stricter emission standards, the transport sector in SEA still contributed as much as 38% to BC emissions in 2015 [39]. There are expected variabilities in the emission contributions in various SEA cities. In Jakarta, Indonesia, for example, the transport sector contributed as much as 46% to the PM_2.5_ emission load in 2015, with a significant share from heavy-duty vehicles [41]. In Bangkok and the surrounding provinces, the total PM_2.5_ emissions from transport were 25 Gg in 2016, accounting for 59% of the total PM_2.5_ emissions from all the sources in the domain [42]. Likewise, mobile sources have the greatest share of the air pollution emissions in Metro Manila, Philippines, accounting for 88% [43]. What is also notable in the transportation environment of the SEA region is the existence of unique public utility vehicles, such as the bajaj in Indonesia; the tuk-tuk found in Thailand, Cambodia, and Laos; and the jeepney in the Philippines. Jeepneys emit up to seven times more BC than light-duty vehicles and contribute more than 60% to the BC emissions in Metro Manila [44]. This estimate was determined using a combination of ambient aerosol measurements with state-of-the-art instrumentation (Multi-Angle Absorption Photometer), fleet information, and a street pollution dilution model. In Bangkok, Thailand, the exposure levels of tuk-tuk drivers to PM_2.5_ were determined for the rainy and dry seasons. These personal exposure estimates were quantified using cascade impactors, which were installed near the drivers [45].

The reports in the scientific literature on the major contribution of transportation sources to ambient PM_2.5_ in the region and the contributions of unique SEA transportation modes highlight the need for more in-depth investigations for better air quality management to reduce health risks. To date, most of these studies have utilized emission inventories, conventional filter-based methods, and sophisticated instrumentation that require considerable resources and infrastructure. On the other hand, the development of LCPMS that can collect high-time resolution data in near real-time provides opportunities for innovative applications [46]. For example, the contribution of sedans to ambient PM_2.5_ was quantified using traffic camera recordings and the AS-LUNG-O [47]. Another study quantified the contribution of different traffic situations to ambient PM_2.5_ using the same LCPMS. Stop-and-go traffic and passing vehicles contributed 4.38 µg/m^3^ and 3.31 µg/m^3^ to ambient PM_2.5_ levels in a mountain community in Taiwan, respectively [9]. Simultaneous measurements using LCPMS in different microenvironments also provide estimates of the contribution of transportation sources to ambient PM_2.5_. In India, a smart personal air quality monitoring system was used to assess the PM_2.5_ levels in a busy traffic site (22.7 ± 8.45 µg/m^3^) and an urban background site (9.3± 5.75 µg/m^3^) [48]. Aside from being able to collect high-time resolution data, LCPMS are also small, light, and portable. These properties make them ideal to use for a variety of applications, such as in evaluating how PM_2.5_ concentrations change with varying pedestrian heights near a roadway [49]. The study used Sensirion SPS30 and Panasonic PM_2.5_ sensors worn at heights of 150 cm and 80 cm from the ground level, respectively. Their results show that PM_2.5_ levels are reduced with increased pedestrian heights. Another novel application of LCPMS was in measuring the three-dimensional distribution of air pollutants aboard a drone. This setup was used to measure the three-dimensional distribution of PM_2.5_ concentrations along a tunnel in Taiwan using two kinds of sensors previously calibrated against Taiwan EPA instruments [50]. The measurements were also used to validate the results of CALINE4 simulations that were aimed at providing air quality forecasts for the area. Results showed that PM_2.5_ at heights of 9.0, 7.0, 5.0, and 3.0 m were 45–48, 30–35, 25–30, and 50–52 µg/m^3^, respectively. The aforementioned applications of LCPMS in evaluating contributions from the transportation sector, particularly in the SEA region, demonstrate their potential to supplement traditional source evaluation methods.

#### 3.1.3. Asian-Style Cooking and Street Cooking

Fuel combustion for daily food preparation is one of the main causes of air pollution in developing countries. This activity releases an array of harmful pollutants, largely due to the incomplete combustion of solid fuels (coal, charcoal, wood, agricultural residue, animal dung cakes) and kerosene in low-efficiency cookstoves. Globally, around 2.6 billion people still rely on solid fuels for cooking in their houses [51]. Almost half of the population is exposed to smoke from cooking with biomass fuel. This exposure causes both short-term impacts, such as coughing, and long-term impacts, such as lung cancer, that lead to mortality and morbidity [52]. In SEA, biomass fuel and biomass-derived fuel (charcoal) provide 78% of the total energy for domestic cooking [53]. Poor households rely more on solid fuels for cooking, hence posing high exposure risks to underprivileged women and children. Yet, the emissions from cooking activities originate not only from the fuel combustion itself but also from the food being cooked, such as fumes from meat grilling [54]. Different cooking methods also generate different amounts of PM_2.5_. Asians have a variety of cooking methods, such as stir-frying, deep stirring, etc., that all increase PM_2.5_ levels during cooking periods [55].

Additionally, in SEA countries, commercial cooking in open streets or street cooking is an important emission source because many locals and tourists prefer street foods. A few articles reported the risk factors of street food vendors by using the points of hazards of the food served, but unfortunately, the emissions from the type of fuel used for cooking food were largely neglected [56]. Although household cooking activities would cause indoor air pollution in the first place (but eventually will be dispersed to pollute the ambient air), street cooking has its emissions directly released at breathing level, causing significant exposure in populated cities. In cities such as Bangkok, Thailand, street cooking activities are observed intensively, especially in busy arterial streets where offices and houses are located. The same situations are found in Malaysia, Myanmar, Indonesia, and Taiwan. Street cooking is a common practice in SEA but an understudied PM_2.5_ source.

High-time-resolution data of air pollution associated with the cooking activities in different indoor and outdoor microenvironments are necessary not only for exposure assessment but also to demonstrate to citizens how the exposure level changes when changing the type of fuel and the stove used for cooking. Such information is essential for their choice of clean cooking systems. However, the monitoring of the pollutants emitted from cooking both in homes and in the streets requires large resources, especially for the high mobility of the street food vendors. With the flexibility of installation at locations without electrical outlets, solar-powered LCPMS can be employed at a high density to monitor PM_2.5_ emissions and the exposure associated with Asian-style cooking and/or street cooking to raise the awareness of all stakeholders [9]. Thus, the use of LCPMS will be most appropriate to provide evidence-based information for the faster intrusion of clean cooking fuel and facilities, both for policymakers and people at the grassroots. Moreover, the monitoring near Asian-style cooking and street cooking may provide essential emissions data to revise emission inventory for improving the PM_2.5_ modeling results. Even though the use of LCPMS for monitoring PM_2.5_ from cooking activities is not intensive in SEA, LCPMS application does have great potential in assessing Asian-style cooking and street cooking for scientific breakthrough and real-world behavior change.

#### 3.1.4. Incense Burning

Incense burning is one of the most peculiar religious and cultural customs in Asian countries such as Vietnam, Taiwan, and Myanmar; it is also a common PM_2.5_ emission source in these countries. Incense burning is particularly regarded as a major indoor PM_2.5_ source, resulting in PM_2.5_ levels that are many times higher than ambient PM_2.5_ levels [3]. Consequently, Asian people may be regularly exposed to high levels of PM_2.5_ during burning periods [7]. This certainly causes significant health risks for Asian people at any age [33,57].

LCPMS have been considered a feasible solution for monitoring PM_2.5_ from incense burning [58]. However, only a few studies have applied LCPMS to assess incense burning in Asian countries. For example, some studies conducted in Taiwan with AS-LUNG showed that PM_2.5_ exposure concentrations from incense burning gradually decreased from personal, household, to community levels [7,13,14]. In addition, at the household level, a study in Hanoi, Vietnam, showed that incense burning caused a spectacular increase in indoor PM_2.5_ concentrations; the indoor PM_2.5_ concentrations at homes that used incense sticks increased by 61.6% compared to the homes that did not, which was determined using an AirVisual Pro Monitor (IQ Air, Switzerland) [59]. Additionally, the high concentrations of PM_2.5_ due to incense burning were found during the Lunar New Year holidays in another study in Hanoi, Vietnam, using Panasonic PM_2.5_ sensors (Panasonic Corporation, Japan) [33]. These studies demonstrated that incense burning caused high personal PM_2.5_ exposures and high PM_2.5_ levels in households and communities [13,59].

Overall, scientists could take advantage of LCPMS to study PM_2.5_ emissions from incense burning in SEA countries that only have limited air-pollution-monitoring resources. In addition, it is worth looking at incense-burning activities in religious places of worship, such as temples and pagodas, where the PM_2.5_ emission concentrations may be very high. People working or living in these places may be the highest risk group exposed to PM_2.5_ from incense burning. In addition to assessing daily PM_2.5_ exposure in previous studies, it is extremely useful to evaluate PM_2.5_ exposure on holidays or special days when burning incense activities dramatically increase, such as the Lunar New Year, the first days of the lunar months, and full moon days. The behavior-change tactics to avoid high exposure under these conditions, such as opening windows, using one incense stick per visit, or avoiding downwind spots, could be explored with LCPMS for alternatives to reduce health risks. Thus, the in-depth investigation with LCPMS allows scientists to understand PM_2.5_ emission strength under different real-world conditions and enhance the public’s awareness of practical options of lowering PM_2.5_ exposures while observing traditional rituals.

#### 3.1.5. Open Waste Burning

The World Bank estimates that one-third of waste generated is not safely managed. Open waste burning causes the release of PM_2.5_ and BC [60]. The WHO estimates that the projected waste will reach 3.4 billion tons every year as the global population increases to 10 billion by 2050 [61]. Open burning of municipal solid waste (including dried leaves) releases PM_2.5_ and a range of harmful gases into the surrounding environment, impacting ambient air quality and human health. The open waste burning practice is prohibited in developed countries by law; however, SEA countries still continue this practice due to a lack of awareness and inadequate waste management. Therefore, there is an imperative need to enhance the awareness of decision makers and communities on this issue. There are limited studies assessing air pollutants emitted from open waste burning using questionnaire surveys, emission inventory, and simulated open burning experiments in SEA. One study in Thailand estimated that open burning emitted PM at the rate of 16.5 kt/year [62]. One study simulated the open burning of municipal solid waste, measured toxic substances released, and reported that emission factors of PM_2.5_ and BC were 6.4 ± 5.1 and 1.1 ± 0.7 g/kg, respectively [63]. Another study in Vietnam revealed that the burning of household solid waste at the disposal site released various kinds of air toxins, including PM_2.5_ (159 t/yr.) and BC [64]. These studies showed that open waste burning is a regional issue in SEA. However, data were not available for open waste burning in the emission inventory in most countries in SEA.

Two studies in Myanmar have used LCPMS to assess PM_2.5_ levels due to open waste burning. One study was conducted in the final waste disposal site throughout three seasons in Mandalay, Myanmar, in 2018–2019 using AS-LUNG-O. The PM_2.5_ monitoring at the final waste-disposal site (both upwind and downwind) was conducted for 7 consecutive days in selected months of the summer, rainy, and winter seasons. Ambient PM_2.5_ concentrations monitored in the summer (24-h mean: 194 ± 19 μg/m^3^) were the highest compared to other seasons due to long-lasting open waste burning. Even though there were still open-burning practices observed in the rainy season, PM_2.5_ concentrations (24-h mean: 61 ± 11 μg/m^3^) were reduced due to the wash-out effect. In addition, PM_2.5_ concentrations (24-h mean: 68 ± 11 μg/m^3^) were significantly reduced during the winter season, when open burning was stopped due to the increasing awareness of its impact. The mean levels of the waste-disposal site in the summer and rainy season were several times higher than the 24 h national air quality guideline (25 μg/m^3^), as well as two to three times higher than the control site where the monitoring was simultaneously conducted [65]. Another study on PM_2.5_ distributions in different townships of Yangon, Myanmar used a Pocket PM_2.5_ Sensor (Yaguchi Electric Co., Ltd., Miyagi, Japan), which was calibrated against a continuous PM monitor (PM-712, Kimoto Electric Co., Ltd., Osaka, Japan). The results revealed that the open burning of dried leaves in the Kamayut Township is one of the potential sources for the highest range of PM_2.5_ (67.8–281.8 μg/m^3^) [66].

Apart from these studies, until now there have been no similar studies on the ground monitoring of PM_2.5_ at waste-disposal sites using both LCPMS and conventional air instruments in SEA countries. Therefore, there is a research gap that could be filled by applying LCPMS for waste burning in this region to highlight environmental and public health threats. PM_2.5_ monitoring with LCPMS at the open waste burning sites at a lower cost can make air quality monitoring possible in many more locations to enhance the awareness of decision makers and communities. Strategies could then be formulated to phase out this practice in SEA.

#### 3.1.6. Fuel Combustion for Brick Manufacturing

Emissions from brick kilns are a serious concern in many countries, especially in SEA [67,68]. Emissions from brick kilns contributed up to 58% of PM_2.5_ and 31% of BC concentrations in Dhaka, Bangladesh [67,69]. Brick production is also one of the main industrial activities in Kathmandu Valley, which is the second-largest contributor to air pollution in Nepal [70]. Nepal produced 1.8% of the total bricks in South Asia [71]. The estimated emission factors of PM_2.5_ and BC from fixed chimney Bull’s trench kilns (FCBTKs) in Nepal were 3.8 ± 2.6 and 0.6 ± 0.2 g/kg, respectively [71]. Likewise, brick industries are the third-largest user of coal in India, with more than one hundred thousand brick kilns producing two hundred fifty million bricks [72]. About 0.94 million tons/year of PM were emitted from brick kilns in South Asia. About two hundred thousand brick kilns are located in and around urban areas of Pakistan and, hence, contribute to the poor air quality. Pakistan has been focused on reducing BC emitted from brick kilns [73].

Several technologies are used in brick manufacturing, such as fixed chimney kilns (FCKs), zigzag kilns, hybrid Hoffman, vertical shaft brick kilns (VSBKs), and tunnel kilns; all of them emit pollutants to some extent [74]. FCKs have the highest emissions; others are relatively modern technologies with higher combustion efficiency. Many countries are replacing FCK technology with modern technology [72]. Emissions from brick kilns have already been reported in many previous studies with research-grade instruments (e.g., [72,75,76]). However, the application of LCPMS in the emission estimation of brick kilns is still very limited. PM_2.5_ was measured with a monitor (Aerocet, USA and Aeroqual, NZ) and filter-based BC and brown carbon at 18 brick kilns with three different technologies in Bangladesh [74]. PM_2.5_ concentrations were the highest in FCKs, followed by zigzag and Hoffman kilns. LCPMS will be a promising tool for emission estimation and characterization, and results can be brought to policymakers to formulate control measures.

As reviewed above, regional biomass burning, tailpipe emissions from various locally made vehicles, Asian-style cooking and street cooking, incense burning, open waste burning, and fuel combustion for brick manufacturing are important but understudied emission sources in the SEA. A better characterization of these sources will improve emission inventory, subsequent PM_2.5_ regional modeling, and source control prioritization as well as enhance the awareness of the general public. With new scientific tools such as LCPMS, breakthroughs in scientific understanding, pollution control, and resulting health risk reduction could be a great contribution to the SEA scientific community and civil society.

### 3.2. Publications on Ambient Monitoring and Transport in SEA Using LCPMS

This section is focused on reviewing articles assessing ambient PM_2.5_ or regional transport in SEA using LCPMS. Ambient PM_2.5_ in the eight countries and the impacts of regional biomass burning assessed with LCPMS are summarized in Section 3.2.1. In addition, LCPMS networks established in some countries to assess ambient PM_2.5_ levels are reviewed in Section 3.2.2.

#### 3.2.1. Ambient PM_2.5_ Levels in the Eight Countries Using LCPMS

Table 2 summarizes available publications assessing ambient PM_2.5_ in these eight countries with LCPMS. The LCPMS used and whether they were calibrated are specifically listed. Ambient PM_2.5_ levels are presented when the measurements were taken by the calibrated LCPMS. The following paragraphs briefly comment on the publications for each country in alphabetical order.

In Bangladesh, ambient air quality monitoring with LCPMS is still very limited. AEROCET 531S (USA) was used to measure real-time particulate matter (PM_1_, PM_2.5_, PM_4_, PM_7_, PM_10_, and total suspended particles) at five different locations, both indoor and outdoor, in Dhaka, Bangladesh (Table 2) [8]. Mean PM_1_, PM_2.5_, and PM_10_ concentrations were 46.1  ±  13.4, 76.0  ±  16.2, and 203.9  ±  44.8 μg/m^3^, respectively. The correlation between indoor and outdoor PM_2.5_ (*R*^2^  =  0.42) and ratios (I/O  <  1) suggested that indoor air was affected by outdoor air. They also studied the correlation of PM_1_ concentrations with the lung function efficiency of residents indoors. There were no published articles on the long-term ambient measurements of PM in Bangladesh. However, data of continuous PM_2.5_ measurements with AirVisual devices in Dhaka are available on a website (https://www.iqair.com/bangladesh/dhaka, accessed on 8 November 2021).

In Indonesia, international publications on ambient PM_2.5_ monitoring with LCPMS are limited. Studies using Edimax AirBox AI-1001W V3 and Alphasense OPC-N2 to measure PM_2.5_ at different locations in Jakarta were presented by one conference proceeding [77] and one undergraduate thesis [78], respectively. Indoor and outdoor PM_2.5_ levels were measured at a hospital, and the average outdoor concentrations measured at that hospital ranged from 50 to 65 μg/m^3^ (Table 2), and the ratio between indoor and outdoor was 0.8 [77]. This suggested that indoor concentrations were influenced by outdoor (ambient air) concentrations. The undergraduate thesis showed that PM_2.5_ concentrations measured at the center of Jakarta had an average concentration of 53.7 μg/m^3^ [78]. Since 2018, there have been several monitoring stations in Jakarta operated by the city of Jakarta and other institutions for measuring PM_2.5_ with traditional instruments; however, there are no publications on the long-term trend of PM_2.5_ yet_._ Thus, LCPMS could be used by researchers to obtain PM_2.5_ levels in areas across Indonesia to complement government monitoring stations and provide observations for environmental research.

In Malaysia, the mean PM_2.5_ concentrations were between 10 and 30 µg/m^3^ during normal days using high-volume samplers [86]. The average PM_2.5_ concentration was 19.1 µg/m^3^ in the Petaling Jaya area near Kuala Lumpur using AiRBOXSense, as shown in Table 2 [79]. Compared to those measurements during biomass burning with more than 100 µg/m^3^ [32,33,34,35], this demonstrated the significant impacts of the transboundary transport of biomass burning on ambient PM_2.5_ levels in Malaysia during burning seasons. Therefore, the multiple deployments of LCPMS in downwind locations can be used to assess the biomass burning impacts due to regional transport in the ambient PM_2.5_ levels in areas without EPA stations in SEA.

In Myanmar, ambient PM_2.5_ was monitored in seven townships of Yangon City, Myanmar, using a calibrated Pocket PM_2.5_ Sensor (Yaguchi Electric Co., Ltd., Miyagi, Japan) in January 2018 (Table 2). The results showed that the highest PM_2.5_ concentrations were observed in the morning, followed by the evening, and the lowest concentrations were observed in the afternoon in all townships. Among the seven townships, Hlaingtharyar Township had the highest concentrations in the morning (164 ± 52 μg/m^3^) and in the evening (100 ± 35 μg/m^3^). Data from eight tracks in Kamayut Township also indicated that PM_2.5_ concentrations varied between different areas and conditions of the same township at the same time [66]. Additionally, there was one study assessing ambient PM_2.5_ levels at the open waste disposal site located in the Patheingyi Township, Mandalay, Myanmar, in the years 2018–2019 with AS-LUNG-O sets [65]. In the control site without burning, mean levels were 94 ± 10 μg/m^3^ in the summer and 53 ± 2 μg/m^3^ in the winter, respectively. Moreover, the Citizen’s Science and Airbeam Myanmar Project monitored ambient PM_2.5_ levels in six cities in 2017–2018 across the country, including Bago, Maubin, Dawei, Yangon, Loikaw, and Taunggyi; it was found that PM_2.5_ levels ranged from 12.0 to 150.4 μg/m^3^ in these cities with notable temporal and spatial variability, with Yangon and the northern part of Dawei having relatively higher PM_2.5_ levels than others. PM_2.5_ levels were monitored with low-cost AirBeam sensors without a specified calibration [87]. Therefore, the detailed results are not presented in Table 2. Based on these findings, properly calibrated LCPMS are very useful for a country such as Myanmar, which still needs to establish air-quality monitoring networks.

In the Philippines, to date, ambient air monitoring using LCPMS is still at an early stage of development. Many of the published studies have focused on the development of monitoring platforms that are able to measure environmental parameters continuously, remotely, accurately, and with minimal cost. The environmental parameters include PM_2.5_ and PM_10_, as well as trace gases (CO_2_ and CO), ambient temperature, relative humidity, and atmospheric pressure. Since many of these studies are still in the development and testing stages, PM_2.5_ measurement durations ranged from a few hours [80,81,82] to a few days [88] to evaluate the sensor’s performance in a variety of locations (e.g., remote, roadside, and on-road) and situations (e.g., stationary, in motion). Therefore, the reported PM_2.5_ levels may not represent ambient levels and are not presented in Table 2.

For Taiwan, there were 27 papers related to LCPMS applications in the Web of Science database up to September 2021. Most of them used different data techniques trying to adjust the data of sensors in the national sensor network (e.g., [89]); these sensors were not calibrated by the manufacturers. Some papers did not mention the necessary calibration for sensors (e.g., [90]), and some mentioned calibration without specifying the calibration methods (e.g., [91]). The application of the sensor network is reviewed in Section 3.2. Here, we present ambient PM_2.5_ levels in Taiwan taken by AS-LUNG-O in three publications. During the monitoring period of 2017–2019, mean levels were below 20 μg/m^3^ in Taipei and Central Taiwan in July; PM_2.5_ in December were higher than those in July in Central Taiwan, with mean levels of 29.2 μg/m^3^ (Table 2) [7,9,47]. In addition to assessing ambient PM_2.5_, these publications also identified hot spots and quantified the incremental PM_2.5_ contributions from different sources within communities where people are in close contact daily. The peak exposures that occurred repeatedly may lead to significant health impacts. The quantification of incremental PM_2.5_ contributions due to these sources can serve as scientific evidence to urge the authorities to target these community sources with stronger regulations and enforcement.

In Thailand, there are quite a few articles using LCPMS in different regions published in local journals using the Thai language. There are only two international publications providing information on ambient PM_2.5_ monitoring results, both in northern Thailand. Ten sensor nodes (Plantower PMS7003) were deployed in the Mae Sot district, Tak province, located at the borderline of Thailand and Myanmar during March–April 2018 to investigate the movement of biomass burning plumes [34]. The results showed that during an intensive biomass burning period, the 24-h PM_2.5_ reached a maximum of 280 μg/m^3^ (Table 2). During a low burning period, the levels were generally low, with a minimum of 13 μg/m^3^. LCPMS can be used not only to monitor the real-time PM_2.5_ but can also be an effective tool for gaining insights into plume dispersion. Another paper reported PM_2.5_ measurement using a PMS3003 attached to a drone in the Nan province [83]. The readings of PM_2.5_ levels along the drone flight track varied from below 5 μg/m^3^ to about 37 μg/m^3^. The monitored data from LCPMS were compared against the tapered element oscillating microbalance (TEOM), with an R^2^ of 0.66 and 0.52 for PM_2.5_ and PM_10_, respectively. Additionally, there is one paper indicating the benefits and potential of using LCPMS to support policies for air quality management in Thailand [92].

In Vietnam, most of the studies using LCPMS have been conducted in the last five years, and mainly in the two largest cities, Hanoi and Ho Chi Minh City. These studies focused on assessing the effectiveness and feasibility of LCPMS [85,93], applying LCPMS in small pilot studies to monitor the ambient PM_2.5_ levels [85,93] or to verify an air-quality forecasting system of PM_2.5_ [84], in consideration of the impacts of meteorological variables and long-range transports [32,33] (Table 2). These studies all applied calibrated LCPMS but with different reference instruments or calibration approaches. In general, there are three crucial issues in the current usage of LCPMS in Vietnam. First, the national air pollution monitoring networks have not been established to create high-quality data which may be used to calibrate LCPMS. Second, there is a lack of national guidelines for LCPMS applications for different purposes, such as the design and development of LCPMS, improving the modeling of spatiotemporal variations for PM_2.5_ mapping, or assessing personal PM_2.5_ exposure. Finally, the data scarcity of PM_2.5_ measurements in Vietnam underlines the importance of using LCPMS for PM_2.5_ monitoring.

Based on the above evaluation, LCPMS have been applied in assessing ambient PM_2.5_ to a certain extent in all these countries, despite limited international publications for some countries. Calibration for these LCPMS is still an issue that needs to be resolved. Compared to traditional research-grade instruments, LCPMS can particularly make contributions in assessing regional PM_2.5_ transport with multiple deployments set along the potential transport paths with much lower costs; they may serve to compel the authorities towards quick responsive actions and to warn local residents with some lead time in areas without EPA stations. Vertical PM_2.5_ profiles may also be assessed with LCPMS in drones to investigate transport/dispersion mechanisms. Moreover, for routine monitoring of ambient PM_2.5_ levels, LCPMS can be deployed in high-population-density areas without EPA stations to complement current air-quality monitoring networks to identify local hot spots within residential communities and enhance citizens’ awareness of the local air quality. Furthermore, there are no publications using LCPMS combined with chemical analysis for PM_2.5_ compositions in these eight countries. Composition analysis is crucial in evaluating the relative contributions of various sources; targeted source control strategies could be formulated afterward. These aforementioned research gaps, which could be filled by the applications of LCPMS, are summarized in Section 3.5.

#### 3.2.2. LCPMS Networks in SEA

Some countries have established LCPMS networks to monitor ambient PM_2.5_ levels in areas of high environmental concern. The LCPMS network is loosely defined here based on the self-claim of the publications; the minimum number of LCPMS was five of those networks in the following review. Since data quality is crucial in scientific works, the following discussion on LCPMS networks will specify whether the network observations have been calibrated with traditional laboratory and field evaluations or newly developed techniques in data science.

LCPMS networks have been set up in Asia for assessing ambient PM_2.5_ levels and identifying/evaluating PM_2.5_ hot spots. In Delhi, India, an LCPMS network of 22 APT-MAXIMA sets (Applied Particle Technology, Inc., St Louis, MO, USA) was established to measure PM_1.0_, PM_2.5_, and PM_10_ since 2019 without a specified calibration method [35]. In Hanoi, Vietnam, another LCPMS network (FAirNet), including 17 sites, measures PM_2.5_ with sensors (PMS7003, Plantower, Beijing, China) calibrated in situ with the instruments of EPA stations with an R^2^ of 0.41 and RMSE of 26.13 μg/m^3^ [93]. In addition, a temporary network of seven Pocket PM_2.5_ sensors (Yaguchi Electric Co., Ltd., Miyagi, Japan) was deployed to measure PM_2.5_ in Yangon City, Myanmar [66]; these LCPMS were calibrated against a PM monitor (PM-712, Kimoto Electric Co., Ltd., Japan). Moreover, a network of 11 AS-LUNG-O sets, calibrated by a GRIMM instrument in the laboratory with an R^2^ of 0.95, was established for assessing PM_2.5_ temporospatial variations and quantifying incremental source contributions of markets, temples, and fried chicken vendors to ambient PM_2.5_ levels [9].

LCPMS networks were also used to evaluate PM_2.5_ reduction during lockdown periods. In Sri Lanka, a network of five sensors (PMS1003, Plantower, Beijing, China) was deployed to measure PM_2.5_ in urban and residential environments [94]; PM_2.5_ sensors were calibrated against a TEOM. They found that the reductions in PM_2.5_ were 10.2%–52.4% during COVID-19 lockdown periods. In Malaysia, a network of five OPC-N2 (Alphasense Ltd., Great Notley, Braintree, UK) was deployed to measure PM_2.5_ [79]; LCPMS were calibrated using a research-grade instrument with a correlation coefficient of 0.71. A general reduction in PM_2.5_ levels was observed in four of these five sites during the lockdown period.

Aiming for citizen science and environmental education, a network that consists of more than 2500 LCPMS (AirBox, with PMS3003 and PMS5003, Plantower, Beijing, China) has been established in Taiwan to measure PM_2.5_ since 2017, with the cooperation of scientists, citizen groups, commercial companies, and government agencies [95,96]. These LCPMS were not calibrated by the manufacturers. With this scale of deployment, traditional laboratory or field calibration is impossible. Post-deployment calibration techniques in data science were developed to improve the data quality of this network. For example, a generalized additive model was used to estimate the correction of two LCPMS comparing observations from EPA stations to improve RMSE from 15.6–31.3 μg/m^3^ to 4.9–9.7 μg/m^3^ [97]. Machine learning was applied to obtain corrections for 39 LCPMS sets with an RMSE improvement from 16.2 to 5.0 μg/m^3^ [47]. Although the accuracy and precision of LCPMS might not reach the level of those research-grade instruments, the LCPMS network with the aforementioned correction has made a great contribution, showing the fine temporospatial variations of PM_2.5_ as never before. For instance, this LCPMS network has shown that PM_2.5_ levels decreased by 3.7% and 10.6%, respectively, covering most areas in northern and southern Taiwan during 2020 pandemic periods even without lockdown measures in Taiwan [91].

In addition, advanced techniques in data science were used to detect malfunctioned LCPMS in this network. An anomaly detection framework (ADF) was developed with statistical and machine-learning methods to identify abnormal sensors among more than 1500 LCPMS deployed in 20 cities [98]. An advanced method was further developed to enhance the detection rate of faulty sensors from 38% by ADF to 97%, and that of incorrect data from 0.8% by ADF to 24% [96]. The detection of malfunctioned LCPMS and erroneous data also contributes to improving the data quality of this national LCPMS network.

In summary, sensor networks have been established for different purposes. Currently, networks operated by environmental scientists mostly use LCPMS calibrated in the laboratory or field against reference instruments to achieve accuracy levels close to research-grade instruments for research purposes. The cost, laborpower, and resources needed for calibration limited the number of LCPMS sets in the network, with the maximum number reaching 20–30 in the aforementioned environmental publications. On the other hand, the network in Taiwan supported by citizen groups and governmental agencies has thousands of LCPMS without calibration beforehand. Aiming for citizen science and environmental education, this network without calibration can show hot spots and temporal trends; in addition, quickly-growing techniques in data science may help to enhance data quality greatly. The related publications were mostly written by data scientists trying different data-handling techniques. For the purpose of this review, since environmental scientists in SEA cannot afford to maintain such a large LCPMS network in reality, the following discussion on environmental research gaps filled by sensor networks is under the assumption of operating a network with less than 30 LCPMS sets.

### 3.3. Publications on Exposure Assessment in SEA Using LCPMS

This section is focused on reviewing articles assessing personal PM_2.5_ exposures of the general public and certain high-exposure or susceptible subpopulations in SEA using LCPMS. Publications on assessing 24-h personal PM_2.5_ exposure using LCPMS in SEA are reviewed in Section 3.3.1. Publications pinpointing the activities associated with peak PM_2.5_ exposures in SEA using LCPMS are in Section 3.3.2.

#### 3.3.1. The 24-h Personal PM_2.5_ Exposure

Several studies used LCPMS to assess 24-h exposures of the general public or a certain subpopulation with specific characteristics. One study assessed personal PM_2.5_ exposure levels of travelers in SEA using Plantower sensors [99]. Their exposure levels depended on the microenvironments they stayed in and the direct PM_2.5_ sources they encountered, e.g., 32.8 µg/m^3^ in the port/station and 29.6 µg/m^3^ in the cafe/pub/restaurant. The maximum exposure (1142 µg/m^3^) happened in an outdoor cafe/pub/restaurant with tobacco smoke, and the second-highest (525 µg/m^3^) was also in a cafe/pub/restaurant, but indoors, with cigarettes and hookah smoke [99]. However, the mean values of PM_2.5_ exposures were one order of magnitude lower; these travelers experienced mean PM_2.5_ levels of 9.6, 14.7, 16.5, 10.9, and 16.0 µg/m^3^ in Thailand, Cambodia, Singapore, Taiwan, and Hong Kong, respectively. This demonstrates the importance of assessing peak PM_2.5_ exposures, which typically are much higher than the mean values. Due to the limitation of the devices used, previous studies used 24-h integrated filter samples to assess mean personal PM_2.5_ exposure (e.g., [100]) or used a much heavier real-time instrument than LCPMS to assess peaks [101]. Those with filter-based measurements cannot detect the much higher peak exposure, whereas the much heavier personal monitors give extra burdens to the subjects who may change their behaviors. The lightweight and easy-to-carry features of LCPMS can detect peak PM_2.5_ exposures without giving heavy burdens to the subjects.

Additionally, several studies assessed 24-h PM_2.5_ exposures within certain countries. Daily PM_2.5_ exposures of 50 subjects were assessed using AS LUNG-P in Bandung, Indonesia [12]. The 30-min personal PM_2.5_ exposures were 29.9 ± 22.3 µg/m^3^ in the wet season (Nov–Jan) and 30.8 ± 13.8 µg/m^3^ in the dry season (July–Sept). In addition, personal PM_2.5_ exposures of 26 nonsmoking adults were studied in Taiwan using AS-LUNG-P at different indoor and outdoor microenvironments, with a 5-min average of 11.2 ± 10.9 µg/m^3^ [13]. In Yangon, Myanmar, personal PM_2.5_ exposures of 15 housewives and 15 university female teaching staff members were assessed to evaluate a small and lightweight LCPMS (Pocket PM_2.5_ Sensors, Yaguchi Electric Co., Ltd., Miyagi, Japan) [102]. They reported that the average 24-h PM_2.5_ exposure levels of housewives and female university teaching staff were 16.1 ± 10.0 µg/m^3^ and 15.8 ± 4.0 µg/m^3^, respectively. Their study demonstrates the successful use of GPS-attached Pocket PM_2.5_ sensors for mobile sensing and continuous measurement. In Seoul, Korea, the personal PM_2.5_ exposures of 47 asthmatic children were investigated with an emphasis on comparing the accuracy and effectiveness of the real-time personal monitoring using LCPMS (PICO, model: PMM-130) to those obtained from monitoring stations [103]. They found that average PM_2.5_ concentrations measured by personal monitoring were lower than those measured by the monitoring stations, especially when the individuals were indoors, at night, and much further away from the monitoring station. In Chennai, India, high PM_2.5_ exposure levels (44.2 and 48.5 µg/m^3^ in January and October, respectively) at bus stops were reported using LCPMS (Panasonic Corporation) [104]. Moreover, changes in personal PM_2.5_ exposure during the COVID-19 lockdown of four American diplomats in Nepal were studied using APT minima [105]. Personal PM_2.5_ exposures were 50.0% to 76.7% lower during lockdown than before. This demonstrates that large-scale adjustments to mostly fossil fuel-related pollution sources, as well as the regulation of indoor settings and activity patterns, might reduce PM_2.5_ exposure. The above publications used LCPMS to assess personal PM_2.5_ exposures in Asia; more LCPMS applications in SEA will provide actual peak PM_2.5_ exposures for different high-exposure and susceptible populations that have not been investigated in depth before.

#### 3.3.2. Activities Associated with Peak PM_2.5_ Exposures

Taking advantage of the fast-response and easy-to-carry features of LCPMS, the activities associated with peak PM_2.5_ exposures were assessed in this region. For example, PM_2.5_ exposures of 36 nonsmoking, healthy subjects aged 20–65 years in Taiwan were assessed in 2017–2018 with AS-LUNG-P [13]. The top three exposure sources identified were environmental tobacco smoke (ETS), incense burning, and cooking, contributing PM_2.5_ increases of 8.5, 5.9, and 3.5 μg/m^3^, respectively, during 30-min intervals. The activities associated with these PM_2.5_ exposure sources were also identified. Out of all ETS exposure periods, 31.3% and 23.9% were associated with working and commuting, respectively, which were the top two activities associated with ETS exposures. Likewise, the top three activities associated with incense-burning exposures were static leisure activities (26.8%), eating (18.3%), and worshipping (17.1%). Additionally, the top two activities associated with cooking fume exposures were eating (45.8%) and cooking (16.5%). With a similar methodology and AS-LUNG-P sets, the PM_2.5_ exposures of 26 nonsmoking, healthy adults in Taiwan were assessed further in 2018 [7]. In indoor home environments, cooking exposures occurred most frequently. Incense burning activities had the highest mean PM_2.5_ indoor/outdoor (1.44 ± 1.44) ratios at home and, on average, the highest 5-min PM_2.5_ increments (15.0 µg/m^3^) to indoor levels among all single sources. Additionally, in Bangkok, commercial cooking was associated with significant PM_2.5_ exposures, with levels up to 200–300 µg/m^3^, which was determined using a calibrated sensor (Particles Plus Co.) [106].

Personal PM_2.5_ exposures were also assessed for 50 subjects with AS-LUNG-P in Indonesia [12]. Burning mosquito coils were identified as the source of the highest exposure, with peak exposure reaching 241.5 μg/m^3^. Exposures due to mosquito coil burning were higher in the wet season than those in dry seasons. The second highest contributing source was factory smoke, indicating factories within or surrounding residential communities caused high PM_2.5_ exposures to residents. In addition, commuting was identified as one of the major activities for PM_2.5_ exposures [13]. PM_2.5_ exposures during six different transportation modes were assessed with a calibrated LCPMS in Taipei; it was found that driving a car would have 20–30 μg/m^3^ less exposure compared to riding a scooter [107]. Additionally, in-car PM_2.5_ exposures were also assessed in ten different cities, including Dhaka, Bangladesh, using calibrated Dylos monitors under the three conditions of windows open, closed with the fan on, and closed with recirculation [108]. Peak PM_2.5_ exposures in Dhaka were found to reach up to 150 μg/m^3^. Moreover, PM_2.5_ exposures of jeepney drivers in Metro Manila, Philippines, were also up to 150–200 μg/m^3^ with AS-LUNG-P [109], showing significant occupational exposures to traffic emissions. Another occupational exposure example is that of waste-management workers; using an AS-LUNG-P, it was found that PM_2.5_ exposures of workers exposed to open waste burning were up to 150–200 μg/m^3^ in Myanmar [110]. The aforementioned activities were all related to high PM_2.5_ exposures assessed by LCPMS; controlling sources associated with those activities and/or raising people’s awareness to change their behaviors is crucial in reducing their peak exposures and health risks associated with these activities.

As reviewed above, LCPMS have been used in SEA to assess personal PM_2.5_ exposures for certain subpopulations of the general public and to identify microenvironments and activities associated with peak exposures. Due to cost considerations, personal PM_2.5_ exposures have been seldom evaluated in SEA with traditional monitors. Distinctive sources in living environments, as discussed previously, may lead to high levels of PM_2.5_ exposures, which are associated with acute health impacts. LCPMS can detect the peak PM_2.5_ exposure of the subjects who encounter PM_2.5_ sources directly, without changing subjects’ behaviors due to the good features such as being lightweight, easy to carry, and having no noise or vibration. A better understanding of the actual PM_2.5_ exposure levels and associated microenvironments and activities with LCPMS could provide evidence for behavior change tactics to reduce PM_2.5_ health risks in SEA. Identifying sources contributing significantly to peak PM_2.5_ exposure could also assist in prioritizing control strategies for these exposure sources.

### 3.4. Publications on Exposure–Health Evaluations in SEA Using LCPMS

This section is focused on reviewing articles assessing PM_2.5_ exposure–health relationships in SEA using LCPMS. Studies have suggested that both short-term and long-term exposure to PM_2.5_ may cause adverse health effects, particularly to respiratory, cardiovascular, and even brain functions in both epidemiological and toxicological studies [111,112,113,114]. The following review focuses on epidemiological rather than toxicological studies. Works focused on the general public are reviewed in Section 3.4.1, and those focused on high-exposure or susceptible subpopulations are reviewed in Section 3.4.2.

#### 3.4.1. Exposure–Health Evaluation for the General Public

PM_2.5_ epidemiological studies for the general public with a large population size typically use ambient PM_2.5_ from EPA monitoring stations as a proxy for personal PM_2.5_ exposure levels and daily hospital records for health evaluations (e.g., [115]). With LCPMS for PM_2.5_ and novel lightweight bio-sensors for physiological indicators, panel-type epidemiological studies could be carried out with the fine time resolution of PM_2.5_ and physiological indicators to assess acute health impacts of individuals. In addition, detailed individual characteristics (such as education, habits, activity patterns, socio-economic status, etc.) could be acquired in panel studies to adjust for more personal factors in affecting health status compared to cohort studies.

Panel-type PM_2.5_ epidemiological studies usually assess changes in respiratory and cardiovascular functions. Since there are novel bio-sensors available in the market (e.g., Rooti RX, Rooti Labs Ltd., Taipei, Taiwan) to assess heart rate (HR) and heart rate variability (HRV), the following review pays particular attention to those works applying novel bio-sensors for assessing acute changes in cardiovascular functions due to peak PM_2.5_ exposure assessed by LCPMS. Epidemiological studies frequently use HRV as a non-invasive method to assess physiological changes in cardiovascular functions; HRV is regarded as an indicator of cardiovascular mortality [116,117]. Elevated PM_2.5_ exposure has been associated with changed HRV or increased HR using traditional samplers [118], nearby air quality networks [119], and traditional portable monitors [120]. With LCPMS, close-to-reality PM_2.5_ exposure will be obtained, leading to more accurate estimates of exposure–health relationships [121].

The application of LCPMS in exposure–health evaluation is in the infancy stage in Asia. There are three panel-type epidemiological studies in Taiwan using AS-LUNG-P in combination with a novel bio-sensor for HRV (Rooti RX). One study recruited 36 healthy, nonsmoking subjects aged 20–65 years all over Taiwan who were wearing AS-LUNG-P and Rooti RX for 2–4 days in both the summer and winter. At PM_2.5_ exposures of 12.6 ± 8.9 μg/m^3^, significant HRV changes were found right after exposure. After adjusting for confounding factors, the standard deviations of all normal to normal intervals were reduced by 3.68% (95% confidence level (CI) = 3.06–4.29%), and the ratios of low-frequency power to high-frequency power increased by 3.86% (CI = 2.74–4.99%) for an interquartile range of 10.7 μg/m^3^ PM_2.5_, with impacts lasting for 4.5–5 h [13]. Another study recruited 35 healthy, nonsmoking adults living in an urban community in northern Taiwan, and exposure–health evaluations were carried out in two seasons. The mean PM_2.5_ concentrations were 13.7 ± 11.4 μg/m^3^. An increase in PM_2.5_ concentrations of one interquartile range (8.7 μg/m^3^) was associated with a change of −1.92% in high-frequency power and 1.60% in the ratio of low-frequency power to high-frequency power [14]. The third study compared exposure–health relationships obtained from Rooti RX and smartwatches; it was found that the health damage coefficients obtained from smartwatches (0.282% increase per 10 μg/m^3^ increase in PM_2.5_) showed the same direction, with a difference of only 8.74% in magnitude compared to those obtained from certified medical devices [122]. It showed that observations from smartwatches (without certification) had greater variability; however, the easy-to-carry smartwatches enable subjects to carry them for more days than the Rooti RX. This advantage of obtaining a large sample size provided nearly equivalent exposure–health relationships of the studied subpopulations, demonstrating the applicability of smartwatches. Thus, smartwatches are alternatives for SEA scientists to lower the expenses of such panel studies. The above works were conducted by the Taiwanese research group on the Hi-ASAP team. The methodology and technology transfer to other research groups could facilitate the application of LCPMS in assessing PM_2.5_ exposure–health relationships in SEA.

#### 3.4.2. Exposure–Health Evaluation of High-Exposure or Susceptible Subpopulations

To assess individual PM_2.5_ exposures and exposure–health relationships of high-exposure or susceptible subpopulations, most previous panel studies were carried out with traditional instruments in controlled and/or specific microenvironments using heavyweight laboratory instruments; additionally, Holter monitors were used to assess HR and HRV for cardiovascular functions (e.g., [119,123,124]). With previous wearable (but not low-cost) monitors, works extended to commuters and walkers in urban and residential settings to assess the relationships between ambient PM_2.5_ levels and HR responses [120,125]. The availability of smaller and lighter LCPMS will likely stimulate scientists to assess exposure–health relationships of the high-exposure or susceptible subpopulations that are understudied so far.

Here, the subpopulation of professional and amateur athletes was used as an example. Amateur athletes are people with regular outdoor exercising habits. They may experience high PM_2.5_ outdoors during exercise. This group was selected due to the following reasons: first, breathing volume per minute is much greater during exercises; second, a good portion of inhalation during exercise is through the mouth instead of the nose, which does not have a nasal-filtration function; third, inhalation during exercise typically carries pollutants further into the respiratory tract due to a higher airflow velocity (summarized from [126]); fourth, an increasing number of the population participates in exercise training in urban cities as part of a healthy lifestyle, which is made evident by mounting running and cycling events [127]. The self-cleaning function in the respiratory system is an important protective mechanism to remove inhaling particles before reaching the lung tissue. This function is often impaired during endurance sport exercise, so that pollutants that might otherwise be filtered and cleared are instead absorbed [128]. Thus, professional and amateur athletes are both high-exposure and susceptible subpopulations.

However, very few studies are targeted at personal PM_2.5_ exposures and the exposure–health evaluation of the athletic subpopulation. In Germany, a significant negative relationship was found between PM_10_ and the number of passes per match of soccer players [129]. A meta-analysis was conducted on the association between exercise and pollutant exposure [120]. Among the 1946 publications that they searched on PubMed, Cochrane, EMBASE, and Web of Science databases, only 25 studies were identified after screening to evaluate the combined effect of pollution exposure and exercise on health. Ten studies out of the twenty-five addressed PM exposure, and only one of those studies used filter-based personal PM data instead of PM data from ambient stations. These studies on the athletic subpopulation mostly took conventional panel approaches, where they set up a closed or proximal environment with a designated or monitored PM concentration and prescribed exercise routines to measure athletes’ performance with a panel size that was generally less than 25. Using different indicators for sports performance, more than half of these studies concluded impaired exercise performance followed by PM inhalation.

The pollutant levels in the aforementioned studies were somehow different from actual exposure. These studies either used pre-set PM_2.5_ in the laboratory or ambient PM_2.5_ levels from nearby monitoring stations, which were not actual personal PM_2.5_ exposures. As exercise and PM_2.5_ have beneficial and detrimental effects on the cardiorespiratory system, outdoor exercise acts as a double-edged sword in health outcomes. In light of the fact that many people are physically active in an urban environment, more comprehensive studies are needed to understand the short-term physiological responses to outdoor exercise while experiencing PM_2.5_ exposure. Assessing HRV changes in healthy and active professional and amateur athletes may provide clues to the development of cardiovascular and respiratory diseases. Subjects can carry LCPMS without being burdened by the weight; thus, close-to-reality PM_2.5_ exposure and the associated health impacts can be assessed accordingly. Therefore, real-world PM_2.5_ exposure assessment and exposure–health evaluation with LCPMS covering a wider range of environments would be beneficial to understand the health impacts due to PM_2.5_ peak exposure on exercising people who should have higher exposure and health risks than people who do not exercise. This is particularly important in the targeted SEA region, where studies are relatively rare, but PM_2.5_ exposures are thought to be much higher than those in Western cities.

Furthermore, some attempts have been made to assess the PM_2.5_ exposure–health relationship for respiratory functions with LCPMS. For example, one study compared the effects of transportation-related air pollution on respiratory function and symptoms between high and low exposure groups in Ho Chi Minh City, Vietnam, in 2019, using the AirBeam2 (HabitatMap, New York, NY, USA). A total of 100 subjects (including 50 in the high-exposure group (motorcyclists carrying goods and street vendors) and 50 in the low-exposure group (office workers)) was selected and interviewed with a questionnaire for a respiratory symptoms assessment. The average PM_2.5_ concentrations were 28.8 μg/m^3^ and 15.9 μg/m^3^ in the high- and low-exposure groups, respectively. Subjects in the high-exposure group had a seven times higher risk for cough symptoms (odds ratio = 7.27; 95% CI: 2.03–26.05) compared to that of the low-exposure group (*p* = 0.008) [130]. However, this was published in a local journal with an English abstract. Nevertheless, it demonstrated that scientists in SEA have begun to apply LCPMS in exposure–health evaluations for high-exposure subpopulations.

In summary, acute health impacts used to be assessed with daily morbidity or mortality records in cohort studies; recently developed novel bio-sensors allow scientists to assess immediate physiological signal changes right after exposure in panel studies with much finer resolution. In such panel studies, the LCPMS is a much better scientific tool than traditional, heavy monitors to obtain close-to-reality peak PM_2.5_ exposures, since the LCPMS is much less disturbing. With lightweight and easy-to-carry features, LCPMS facilitate subject recruitment in panel studies to assess close-to-reality exposure–health relationships. Especially in resource-limited SEA countries, only a few panel studies have been conducted on PM_2.5_ due to cost considerations. The advancement in both LCPMS and novel bio-sensors provides great scientific tools in evaluating PM_2.5_ exposure–health relationships for understudied high-exposure and susceptible SEA populations.

### 3.5. Research Gaps That Can Be Filled in SEA with LCPMS Applications

To provide convincing scientific evidence to support effective policy formulations for further PM_2.5_ reduction, applying calibrated LCPMS with good data quality is required. This section summarizes priority research questions in SEA that can be addressed by applying the calibrated LCPMS. These are the outcomes of the aforementioned literature review and consensus from participatory workshops in 2019–2021. These research questions are oriented at evaluating certain controllable factors (either targeted source control or designed behavior-change tactics) that can be translated into policy actions. They are listed in the order according to our review framework. The first to fourth sets of research questions correspond to research focuses 1 to 4 in Figure 2; the fifth set of research questions is policy-oriented to protect public health. These five sets of research questions can be simplified as source evaluation, hot spot investigation, peak exposure assessment, exposure–health evaluation on acute health impacts, and short-term standards.

1. What are the source characteristics of the distinctive Asian sources that have been understudied? What are the key factors associated with the sources’ strength?
2. What are the temporospatial variations of ambient PM_2.5_ levels in densely populated areas without monitoring stations? What are the peak PM_2.5_ levels in hot spots within communities that may result in the high exposure of residents?
3. What are the peak PM_2.5_ exposure levels and patterns of the SEA population, especially in high-exposure or susceptible subpopulations? What are the sources, activities, and associated controllable factors causing peak PM_2.5_ exposures?
4. What are the damage coefficients of the exposure–health relationship for respiratory and cardiovascular health outcomes due to peak PM_2.5_ exposures? Are the damage coefficients for the same health outcome different at different PM_2.5_ concentration ranges?
5. Should there be a ceiling value or short-term standard for PM_2.5_ (e.g., 8 h or hourly)? What other considerations need to be included to promote the establishment of such a standard?

The first set of research questions is related to PM_2.5_ emission sources. LCPMS can be used to assess PM_2.5_ emissions from sources, near-source PM_2.5_ concentrations, and the incremental contribution of certain sources to ambient PM_2.5_ levels, as reviewed in Section 3.1. The source characteristics evaluated may include emission strength and temporospatial variations of PM_2.5_ emissions. The size distribution of PM emissions could also be provided since LCPMS with multiple size distributions are currently available in the market (e.g., GSA, Plantower). In addition, if the application of LCPMS could be combined with filter-based sampling and subsequent chemical analysis, the investigation for physico-chemical characteristics of those sources would be more comprehensive.

The targeted sources are understudied distinctive Asian sources, as reviewed above. Evaluating key factors associated with those sources would provide evidence for effective source control or source substitution. The source with the most complicated control strategy is regional biomass burning, which involves traditional agriculture practices and international collaboration. LCPMS networks established along the potential transport pathways to complement EPA monitoring stations could collect substantial evidence of ground PM_2.5_ levels in source and impacted areas for international negotiation and facilitate international collaboration in minimizing the regional impacts. Currently, only limited PM_2.5_ monitoring data in certain areas with official monitoring stations are available; other areas did not have evidence of ground-level PM_2.5_ impacts due to transported regional biomass burning, which may last for several days. LCPMS networks could provide ground-level PM_2.5_ evidence covering more areas with fine resolutions. In combination with short-term PM_2.5_-health impacts, as pointed out in the fourth set of research questions, this solid evidence can be used to support the international negotiation of the impacted countries as never before for arguing better strategies by the source countries. Furthermore, these networks may serve as a warning tool for downwind locations to provide some lead time for certain responsive actions, such as wearing masks to reduce PM_2.5_ exposures and health risks. With LCPMS, PM_2.5_ source strength and transport can be evaluated at multiple locations at a much more affordable cost. Thus, SEA scientists can team up to apply LCPMS and provide ground-level observations to compel authorities to enact better enforcement and enhance the awareness of the affected population for self-protection.

In addition, for vehicle emissions, most PM_2.5_ emission factors of vehicles used in air-quality modeling come from international vehicle brands. LCPMS can be used to provide close-to-reality on-road emission factors, which can be used to quantify city-wide emissions from locally made vehicles to improve PM_2.5_ modeling using methodology demonstrated in several publications [9,47]. For example, with street-level monitoring by LCPMS in combination with vehicle types and traffic volume recorded by traffic cameras and assessed by deep-learning techniques, the contribution of different vehicle types to nearby PM_2.5_ levels were obtained [47], which can be used in street-canyon or dispersion models to back-calculate PM_2.5_ source strengths of different vehicle types. The obtained real-world PM_2.5_ source strength can then be used to update the emissions inventory for subsequent city-wide or regional PM_2.5_ modeling and forecasting. Close-to-reality PM_2.5_ modeling can provide policymakers with better information for effective control strategy formulation targeting high-emitting vehicles. If SEA scientists can collect more evidence of high emissions of certain locally made vehicles, this evidence may urge policymakers to promote electric vehicle policies, which could greatly reduce pedestrians’ PM_2.5_ exposures from vehicles and improve city-wide air quality while maintaining the easy mobility that the SEA population highly values. Substituting current internal combustion engine vehicles will also contribute to climate action.

Moreover, since most of the air quality regulations in SEA follow the examples of Western countries, certain locally important sources are neglected in current regulations. Scientific evidence is needed to compel the authorities to formulate regulations targeting these sources. Currently, the authorities focus on large industrial sources and traffic emissions. Emission sources affecting the air quality of nearby communities and daily residents’ exposures, such as Asian-style cooking and street cooking, incense burning, open waste burning, and fuel combustion for brick manufacturing, may not be included in the control agenda, even though those are important sources contributing to residents’ PM_2.5_ exposures, as shown in the review. LCPMS could be used to quantify their significant emissions, serving as the stepping stone for setting regulations. An emissions inventory could be improved by these real-world assessments; city, regional, and even global air quality modeling could be improved subsequently. Moreover, with convincing evidence, behavior-change tactics to educate people to avoid these sources dotted within communities may reduce residents’ PM_2.5_ exposure and associated health risks.

In terms of cost, traditional instruments used for these source evaluation works are much more expensive than LCPMS, taking EDM-180 and AS-LUNG-O as respective examples. The basic manufacturing cost is around USD 650 for AS-LUNG-O [9]; even if the retail price is three-fold, it is roughly USD 2000. The purchasing cost of EDM-180 (around USD 54,000) is 27 times higher than that of AS-LUNG-O. Thus, for SEA scientists, an LCPMS is a much more affordable research tool for setting up a network. The cost is even higher if the required air-conditioned rooms for EDM-180 are included. Moreover, evaluating the aforementioned community sources, such as on-road traffic emissions or restaurants, is more flexible with an LCPMS, which is small, has solar panels, and can be attached to light poles at street level near those sources, as demonstrated in [9]. Contrarily, the bulky traditional instruments require air-conditioned rooms to obtain stable observations, which restricts the locations of the source evaluations. With multiple sets and long-term monitoring by LCPMS, scientists are able to acquire a much larger sample size of PM_2.5_ observations, which is needed for statistical analysis to obtain source strength or the incremental contribution of these sources to ambient PM_2.5_ levels [9].

The advantage of the low cost is not only manifested in establishing networks without air-conditioned rooms required for traditional instruments; it is also shown in assessing high-emission PM_2.5_ sources in close distances, which may cause contamination to research-grade instruments. For example, cooking fumes may contaminate instruments that require a high cost for cleaning or replacement. The high maintenance of research-grade instruments limits the assessment of cooking emissions. With LCPMS, the low replacement cost allows scientists to design studies for the thorough investigation of such sources without too much financial burden.

The second set of research questions aims to assess the temporospatial variations of ambient PM_2.5_ levels and identify hot spots in densely populated areas without EPA monitoring stations. Most SEA countries have high population densities, as shown in Table 1, whereas the authorities only have limited resources for air-quality monitoring. An LCPMS is a great alternative to assess ambient PM_2.5_ levels in areas without EPA monitoring stations, especially in densely populated areas affected by multiple sources. Not only can an LCPMS assess temporospatial variations of ambient PM_2.5_ levels, but it can also identify/quantify hot spots in residents’ living environments. This evidence can be provided to the authorities for targeted hot spot controls to minimize residents’ PM_2.5_ daily exposures.

This second set of research questions is intertwined with the first set. Wherever there are PM_2.5_ emission sources, such as small factories or restaurants in certain areas, high temporospatial PM_2.5_ variations and hot spots will exist in the respective communities. Identifying hot spots assists in detecting important PM_2.5_ emission sources in that community. Quantifying PM_2.5_ contributions of the previously unknown local sources will greatly contribute to prioritizing control strategies. Afterward, a filter-based chemical analysis can be conducted. Thus, LCPMS can serve as a screening tool for costly chemical analysis. The advantages of LCPMS over traditional instruments in evaluating temporospatial variations and identifying hot spots are the same as those for source evaluation, namely affordability, capability of collecting fine temporospatial-resolution observations with a large sample size, as well as flexibility in terms of power, location selection, and study design.

The third and fourth sets of research questions are focused on personal PM_2.5_ exposure assessments and exposure–health evaluations, respectively. Traditional instruments used for personal PM_2.5_ exposure assessment and exposure–health evaluations in panel studies are filter-based personal samplers, such as PEM (761–203B, SKC Ltd., Blandford Forum, UK), or real-time personal monitors, such as GRIMM. The integrated measurements obtained by PEM suffered from a low temporal resolution; PEM is not capable of assessing peak PM_2.5_ exposure and acute health impacts. Moreover, the fine-resolution GRIMM costs nearly USD 24,000, much more expensive than LCPMS. Taking AS-LUNG-P as an example, the basic manufacturing cost is around USD 270 [13]; assuming retail cost is three-fold, it is roughly USD 800. GRIMM is 30 times costlier than AS-LUNG-P. Furthermore, the heavy weight (2 kg) of GRIMM also restricts its being carried around the waist, slightly distant from the breathing zone for exposure assessment. In addition, the noise, vibration, and apparent outlook of GRIMM often discouraged subjects from adhering to their routine daily activities on the monitoring days.

With advantages such as being small, lightweight, easy to carry, and having no noise or vibration, LCPMS can be applied in personal PM_2.5_ exposure assessments and panel-type epidemiological studies, making it much easier to recruit subjects from the general public and certain high-exposure or susceptible subpopulations than previously. In addition, the lightweight LCPMS will not interfere with subjects’ daily routines. Thus, close-to-reality PM_2.5_ exposures and health impacts could be assessed to obtain actual slopes of exposure–health relationships (PM_2.5_ damage coefficients). Additionally, the fine temporospatial resolutions of actual PM_2.5_ exposure levels obtained by LCPMS can be used to answer research questions, such as peak exposure levels, exposure frequency, exposure duration, the key exposure sources, the associated micro-environment, and activities of the targeted subpopulations. The recommendations for controlling for these important exposure sources and designing for behavior-change tactics can be provided to the authorities accordingly to reduce the PM_2.5_ exposure of the targeted subpopulations.

Furthermore, physiological signals can be detected with newly developed bio-sensors. The damage coefficients of PM_2.5_ for acute health impacts on heart conditions could be assessed accordingly. Certain LCPMS devices such as AS-LUNG-P incorporate other sensors to collect observations which need to be included in the PM_2.5_ exposure–health models. For example, AS-LUNG-P has a motion sensor to detect intensity of human movement, which also affects HRV. Collecting important variables for PM_2.5_ health evaluation with a single device is also a big advantage of LCPMS over traditional instruments.

Moreover, the huge differences in PM_2.5_ levels in SEA provide an excellent testbed to evaluate whether PM_2.5_ damage coefficients are different at different concentration ranges. With a similar methodology to that demonstrated earlier [13,14,122], the damage coefficients of PM_2.5_ exposure-health relationships for the same health outcome (e.g., HRV) can be obtained in different countries for different subpopulations in different PM_2.5_ ranges. Assessing these PM_2.5_ damage coefficients with meta-analysis can provide insights into whether PM_2.5_ exposure-health relationship is linear across different PM_2.5_ levels for the specified health outcomes and whether the PM_2.5_ damage coefficients are similar for different subpopulations. Panel-type epidemiological studies for PM_2.5_ in SEA have seldom been conducted due to the anticipated cost. LCPMS provides a great opportunity for this type of research in SEA, especially in assessing high-exposure and susceptible subpopulations.

The fifth set of research questions is specific to policy. Currently, despite recommended guidelines or mandatory standards for daily and annual limits of PM_2.5_ provided by the WHO and environmental agencies worldwide, there are no guidelines/standards for short-term (8-h or hourly) limits or ceiling values of PM_2.5_ exposures due to the lack of scientific evidence. Given high PM_2.5_ levels in this region, SEA populations require a short-term guideline/standard to reduce their health risks. The scientific evidence obtained in studies targeting PM_2.5_ exposure–health evaluations (the fourth set of research questions) may be able to evaluate the following question: “Should there be a ceiling value or short-term standard for PM_2.5_ (e.g., 8-h or hourly)?” Engaging policymakers earlier in the study design stage may help SEA scientists gain a better understanding of the policy considerations for such a short-term PM_2.5_ standard. Streamlining science–policy dialogs is beneficial for fine-tuning the study design and translating research findings of the aforementioned five sets of research questions into policy actions.

## 4. Discussion

The unique angles and contributions to the international research community of LCPMS applications in environmental health research in SEA are discussed in Section 4.1. The challenges of the current LCPMS application in SEA, as well as possible solutions, are discussed in Section 4.2. The expected outcomes with a transdisciplinary perspective involving stakeholder engagement are discussed in Section 4.3.

### 4.1. Unique Angles and Contribution to the International Research Community

Applying LCPMS to fill the aforementioned research gaps is not only valuable to SEA countries but also contributes to the international environmental health research community. The following paragraphs describe the unique angles and potential contributions.

1. There are distinctive PM_2.5_ sources in SEA, such as outdated practices (e.g., open waste burning, fuel combustion for brick manufacturing), different locally made vehicles (motorcycles, jeepney, tuk-tuks, or others), different types of open biomass burning (forest, peat, rice straw, sugar cane, etc.), different cooking practices (stir-frying, deep-frying, etc.) using a range of solid fuels, and different culture-related activities (incense burning with incense made from different materials) that may cause high PM_2.5_ concentrations in the ambient air and high PM_2.5_ exposures to residents or workers. An international comparison of source characteristics and exposure patterns related to those sources with LCPMS presents a more comprehensive overview of the PM_2.5_ emission features of those sources. These findings can improve emissions inventories and facilitate the improvement of PM_2.5_ modeling and assist in prioritizing the control strategies of the authorities. The improvement of regional emissions inventories will also be beneficial to regional and global aerosol modeling as well as climate modeling, since aerosols have direct and indirect impacts on climate change [131].
2. Episodes due to the regional transport of large-scale open biomass burning impact the source and downwind countries, making this an important international issue in SEA. LCPMS installed in different countries with spatial coverage wider than the standard EPA stations provide more details showing the actual affected levels, areas, and populations. LCPMS networks can provide PM_2.5_ at ground level, where people live, which is superior to remote-sensing images showing aerosol loadings for the whole vertical column. After all, those transported aerosols in altitudes higher than 1000 m may affect visibility but not affect the PM_2.5_ exposure and health of local residents. Thus, from a public health point of view, setting LCPMS networks in SEA is the best way of providing scientific evidence for international negotiations dealing with regional biomass burning. Moreover, early warning systems based on LCPMS networks could be a powerful policy tool, enabling authorities to have quick responsive actions and enhance the self-protection of the general public. This can further serve as an example for African countries to warn about dust storms.
3. Using newly developed LCPMS and bio-sensors with coherence methodology, PM_2.5_ damage coefficients can be obtained for different subpopulations in different SEA countries. Comparing PM_2.5_ health damage coefficients of the same health outcome across different countries with different PM_2.5_ exposure levels can provide insightful knowledge on PM_2.5_ health impacts; this is a fascinating and challenging question and cannot be answered in Western countries with low PM_2.5_. A meta-analysis of the studies conducted in different countries can also support more effective science–policy communication in this region as a whole. The synthesis of these findings can provide policy-relevant recommendations for SEA countries and beyond to reduce PM_2.5_ exposure and health risks wherever needed.
4. Weather patterns in SEA are controlled by the Asian monsoon, which results in significantly different PM_2.5_ levels within a country, i.e., lower PM_2.5_ in rainy seasons and higher PM_2.5_ in dry seasons. On the other hand, under climate change, SEA countries have also experienced more frequent heat waves with extreme temperatures. Both temperature and relative humidity affect human heat stress, which leads to cardiovascular impacts [132]; these weather parameters are confounders of PM_2.5_ health impacts. Currently, certain LCPMS devices, such as AS-LUNG, are equipped with low-cost temperature and humidity sensors to collect PM_2.5_, temperature, and relative humidity simultaneously with a resolution of 15 s, 1 min, or 5 min to evaluate health impacts of PM_2.5_ and weather parameters altogether. Thus, conducting panel epidemiological studies in SEA with LCPMS provides a unique opportunity to assess the synergistic effects of PM_2.5_ (varied in different countries) and heat stress under humid versus dry conditions on cardiovascular functions. Understanding this synergistic effect provides insights into the physiological reactions of human bodies responding to environmental changes. The research findings are particularly valuable under the trend of climate change, since other countries may soon experience heat stress and PM_2.5_ altogether.

### 4.2. Challenges in LCPMS Application

To contribute to PM_2.5_ health risk reduction for local societies in SEA and scientific breakthroughs for the international academic community, two prerequisites of applying LCPMS are the affordability and appropriate methodology associated with such applications. In terms of affordability, even though an LCPMS is low-cost, the application of LCPMS in environmental health research is not as inexpensive as it seems. If only trend analysis is anticipated, uncalibrated LCPMS or calibration with data science (associated with higher uncertainty) may be acceptable. However, since accuracy is crucial in environmental health research, an expensive research-grade instrument and a customized controlled chamber are needed for calibrating LCPMS. Additionally, periodical re-calibration is preferred, since the downtimes for these newly developed LCPMS are mostly unknown. The summation of these costs makes LCPMS applications challenging in SEA.

To lower the barriers in expenses, international collaboration with a technology hub in one country may serve as a solution. Taking Hi-ASAP as an example, Academia Sinica, Taiwan, has served as a technology hub for AS-LUNG. In the initial discussion of Hi-ASAP, the choice of LCPMS was open to any LCPMS with sufficient evidence demonstrating good data quality as nearly equivalent to those from research-grade instruments. By the end of 2019, AS-LUNG stood out as the preferred choice, since three types of AS-LUNG are designed purposely for research; the addition of SD cards for data storage, GPS for locating, and motion sensors for human movements make AS-LUNG convenient scientific tools compared to other commercial products [7,9]. Afterward, the research group of Academia Sinica agreed to provide several calibrated AS-LUNG sets for each participating group to lower the research expense of the Hi-ASAP team.

Additionally, several capacity-building workshops were held by the research group of Academia Sinica for technology and methodology transfer. The ins and outs of applying AS-LUNG properly were shared in these workshops. Moreover, to evaluate controllable factors along the progression of PM_2.5_ emission sources to health impacts with AS-LUNG requires prudent study designs. Further, a cohesive methodology is preferred to carry out international comparisons to enhance the findings’ scientific value. Thus, these workshops aimed to ensure that all research groups of the Hi-ASAP team were on the same page regarding technology, study design, and data analysis methods. This kind of international collaboration can greatly lower the barriers in both expenses and expertise and raise the values of the expected findings. Hi-ASAP’s experience is shared here as an example to encourage more international collaboration on technology and methodology transfer to facilitate LCPMS application in resource-limited developing countries.

### 4.3. Transdisciplinary Perspectives and Stakeholder Engagement

**Figure 3:**
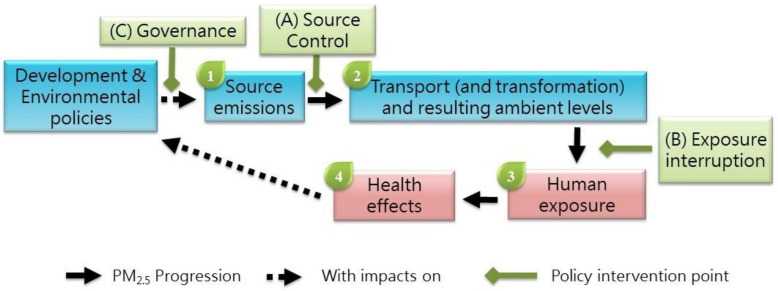
Diagram of policy impacts with intervention points along the progression of PM_2.5_ from sources to health impacts. The intervention points (green arrows) are numbered by letters according to the order of the identified research priorities (1–4), with point A corresponding to priorities 1 and 2, point B to 3, and point C to 4. Black solid arrows indicate the PM_2.5_ progression from sources to health impacts. Black dash arrows indicate policies that impact or are being impacted upon.

The identified research priority targets key controllable factors that can be translated into policy actions that could intervene in the progression of PM_2.5_ from the source to health impacts (Figure 3). With LCPMS, a better characterization of sources and hot spots (research priorities 1 and 2) can assist in prioritizing source control (point A), which reduces ambient PM_2.5_ levels and subsequent exposure and health impacts. Additionally, understanding exposure levels and associated micro-environments and activities (research priority 3) can help to design appropriate behavior-change tactics for the general public and high-exposure and susceptible subpopulations; interrupting exposure pathways with changed behavior (Point B) can effectively reduce their peak and long-term PM_2.5_ exposures and health risks. Moreover, since protecting public health is a noble goal that no policymakers can deny, providing evidence of acute PM_2.5_ health impacts (research priority 4) may compel policymakers to shift the development and environmental policies towards more environmentally friendly directions, which may eliminate or at least minimize emission sources from the beginning (Point C). Pollution-free governance is the best way of reducing PM_2.5_ health risks for SEA and other countries. In resource-limited SEA countries, a properly calibrated LCPMS is a new research tool allowing scientists to assess distinctive Asian sources, hot spots, peak PM_2.5_ exposures, and acute health impacts as never before. Thus, the LCPMS application in PM_2.5_ research holds promise in changing the status quo and truly making breakthroughs in science and policy.

To apply LCPMS for filling these identified research gaps, a collaboration involving environmental scientists, public health professionals, and policymakers is required to investigate the entire PM_2.5_ progression and translate scientific findings into policy actions. In other words, this is transdisciplinary research emphasizing the co-creation of knowledge with stakeholders [133,134]. Thus, support from policymakers is essential for the science–policy translation in PM_2.5_ research with LCPMS in SEA. Engagement with policymakers earlier in the study design stage has been emphasized by Future Earth as an effective way to streamline science–policy dialogue. Future Earth promotes transdisciplinary, solution-oriented, and stakeholder-engaged research to have actual societal impacts with research findings. However, communication with policymakers needs experience and practice, and most scientists have not had such training in their careers. International organizations such as Future Earth could play a crucial role in establishing such a platform for national and international science–policy dialogue. The advancement in international policy may put more pressure on SEA countries as in the case of the climate emergency. Thus, interactions between scientists and policymakers on the international platform can facilitate policy actions within individual countries to a certain extent. Moreover, this platform can also serve as an opportunity for experience-sharing among scientists to learn the art of science communication from each other. By collaboration, scientists would have a better chance to have an actual impact on national and international policies to tackle the PM_2.5_ challenges faced by current SEA countries.

## 5. Conclusions

The advantages of LCPMS include low-cost, lightweight, small, fast-response, easy to carry, easy to operate, with portable power, and no noise or vibration. Certain LCPMS devices can also collect observations of other important variables by incorporating other sensors. This paper reviews current publications applying LCPMS in PM_2.5_ research in SEA under a framework based on the progression of PM_2.5_ emission sources to health impacts, namely source evaluation, ambient monitoring and transport, exposure assessment, and exposure–health evaluation. Special attention was paid to those understudied distinctive Asian sources such as biomass and agriculture open burning, tailpipe emissions from various locally made vehicles, Asian-style cooking and street cooking, incense burning, open waste burning, and fuel combustion for brick manufacturing. As reviewed above, the application of LCPMS in PM_2.5_ research was limited in SEA but with great potential. Scientists in this region have begun to use LCPMS in various studies, showing their capability and intention, even though the publications are not impressive in quantity. Given the severe PM_2.5_ pollution in this region, PM_2.5_ research is urgently needed to provide targeted solutions to lower PM_2.5_ levels and the associated health risks. Applying new technology such as LCPMS in environmental health research with the collaboration of scientists in different disciplines and policymakers with a transdisciplinary perspective provides a great opportunity to change the status quo.

Based on the review and discussion from three participatory workshops with participants from fourteen countries in Asia, the policy-relevant priorities in applying LCPMS in PM_2.5_ environmental health research in SEA are identified as the evaluation for distinctive Asian sources, hot spot investigation, peak exposure assessment, acute health impacts, and short-term standards, taking advantage of their aforementioned good features. The research questions are oriented toward evaluating certain controllable factors that can be translated into policy actions of targeted source control, behavior-change tactics interrupting exposure pathways, or the promotion of pollution-free environmental governance to reduce health risks due to PM_2.5_. These policy-relevant research questions must be resolved with accurate data obtained from the calibrated LCPMS.

Applying LCPMS in PM_2.5_ research in SEA could make significant contributions to local civic society as well as the international scientific community. A better characterization of distinctive Asian sources and hot spots with LCPMS can be used to provide a close-to-reality emission inventory to improve regional and global PM_2.5_ modeling and climate modeling. The acquired knowledge on source strength is also crucial to prioritize local source-control strategies and to devise targeted hot spot controls. Minimizing fossil fuel combustion could have co-benefits on both health and the climate in this post-COVID-19 era. LCPMS networks along the transport pathways of regional biomass open burning could serve as a warning system at high-population locations without governmental stations and could provide lead time to reduce regional health risks. LCPMS networks could also complement official monitoring stations with much lower costs to provide PM_2.5_ levels and to facilitate PM_2.5_ research in SEA. In addition, very few PM_2.5_ exposure and health studies have been conducted in SEA. Better assessments on peak PM_2.5_ exposure levels and acute health impacts with LCPMS covering more areas with more diversified high-exposure and susceptible subpopulations at different concentration ranges could provide in-depth evidence on whether short-term (one-hour or eight-hour) standards are needed for public health protection. The synthesized knowledge can provide policy recommendations for the whole SEA region and other regions to lower global PM_2.5_ health risks. Moreover, the synergistic effect of heat stress and PM_2.5_ can be assessed in these subtropical and tropical countries; this is particularly valuable under the trend of climate change. The hot and humid conditions and wide range of PM_2.5_ levels in this region provide a testbed to evaluate those crucial scientific questions using LCPMS. The barriers of expenses for calibration with expensive instruments and laboratory set-up and the expertise for prudent study design to fill those research gaps can be lowered by international collaboration. Finally, stakeholder engagement is key to successfully translating scientific findings to policy actions for making changes on the ground. The science–policy dialogue could be streamlined if scientists could collaborate and share successful experiences via international platforms. With international collaboration, the knowledge acquired with LCPMS application in PM_2.5_ research in SEA is expected to make significant contributions to SEA populations and the international scientific arena.

## Figures and Tables

**Table 1:** Characteristics of (a) the eight countries and (b) the capital cities discussed in this paper. Data presented are 2019 statistics except for area and PM_2.5_, which are from 2020.

**(a) Country**	**Population** [15] **(Estimate, Thousands)**	**Total Area** [16] **(km^2^)**	**Population Density of the Entire Country** **(Person/km^2^)**	**GD** **per Capita** [17] **(USD)**	**Employment in** **Industry** [18] **(% of Total Employment)**
Bangladesh	163,046	143,998	1132.3	1856	21
Indonesia	270,626	1,904,569	142.1	4136	22
Malaysia	31,950	329,847	96.9	11,414	27
Myanmar	54,045	676,578	79.9	1477	17
Philippines	108,117	300,000	360.4	3485	19
Taiwan	23,774	36,193	656.9	25,941 [19]	63 [20]
Thailand	69,626	513,115	135.7	7807	23
Vietnam	96,462	331,689	290.8	2715	27
**(b) Country/Capital City**		**Population in the Capital City in 2019 (Estimate, Thousands)**	**Population Density in the Capital City in 2019** **(Person/km^2^)**	**Annual Mean of Hourly PM_2.5_** [21] **(μg/m^3^,** **Capital City, 2019)**	**Annual Mean of Hourly PM_2.5_** [10] **(μg/m^3^,** **Capital City, 2020)**
Bangladesh/Dhaka		20,284 [22]	23,234 [22]	83.3	77.1
Indonesia/Jakarta		10,639 [22]	15,900 [23]	49.4	39.6
Malaysia/Kuala Lumpur		7780 [22]	7802 [24]	21.6	16.5
Myanmar/Yangon		5244 [22]	12,308 [22]	31	NA
Philippines/Metro Manila		13,699 [22]	21,765 [25]	18.2	13.1
Taiwan/Taipei		2645 [26]	9473 [26]	13.9	12.6
Thailand/Bangkok		10,350 [22]	6598 [27]	22.8	20.6
Vietnam/Hanoi		4480 [22]	2410 [28]	46.9	37.9

NA: Not available.

**Table 2:** Summary of ambient PM_2.5_ levels in the eight countries obtained with low-cost PM_2.5_ sensors (LCPMS).

Country	Studied Area	Year	PM_2.5_ Levels (μg/m^3^)	Sensor Used	Calibration
Bangladesh	Dhaka [8]	2017	76.0 ± 16.2	AEROCET 531S	Yes
Indonesia	Jakarta [77]	2019	50–65	Edimax AirBox AI-1001W V3	Yes
	Jakarta [78]	2018–2019	53.7 (0–175)	Alphasense OPC-N2	Yes
Malaysia	Petaling Jaya near Kuala Lumpur [79]	Nov 2019–Feb 2020	19.1	AiRBOXSense	Yes
Myanmar	Yangon [66]	2018	Hlaingtharyar, Morning 164 ± 52 Evening 100 ± 35	Pocket PM_2.5_ Sensor	Yes
	Yangon [66]	2018	Kamayut, Morning 91 ± 37 Evening 60 ± 22	Pocket PM_2.5_ Sensor	Yes
	Mandalay [65]	2018–2019	Summer 94 ± 10 μg/m^3^ Winter 53 ± 2 μg/m^3^	AS-LUNG-O	Yes
Philippines	Quezon City, Metro Manila [80]	2017	--	CrowdSSense	No
	Manila and Taguig and Makati Cities, Metro Manila [81]	2019	--	--	No
	Balanga City, Bataan Province [82]	--	--	DSM501A	No
Taiwan	Central Taiwan [9]	2017	July 17.5 ± 8.9; December 29.2 ± 10.6	AS-LUNG-O	Yes
	Taipei [7]	2018	18.4 ± 10.6	AS-LUNG-O	Yes
	Taipei [47]	2018–2019	Location A 17.2 ± 9.1; Location B 10.8 ± 3.9	AS-LUNG-O	Yes
Thailand	Mae Shot, Northern Thailand [34]	Mar–Apr 2018	13–280 (24h)	Plantower PMS7003	Yes
	Nan, Northern Thailand [83]	NA	<5–37 (flight track)	Plantower PMS 3003 (on Drone)	Yes
Vietnam	Hanoi and Thai Nguyen Province [33]	Oct 2017–Apr 2018	Hourly: three sites, 57.5, 54.9, and 53.6	Panasonic PM_2.5_ sensors	Yes
	Ho Chi Minh City [84]	2017	Maximum: 30–34 Minimum: 5–10	Plantower PMS 3003	Yes
	Ho Chi Minh City [85]	Oct–Dec 2018	Sensor 1: 33.86 Sensor 2: 34.16	Plantower PMS 3003	Yes

## Data Availability

Not applicable.

## Appendix A

**Table A1:** Participant list of the Hi-ASAP planning workshop held in Academia Sinica, Taipei, Taiwan on 17–19 May 2019.

	Research Region	Participant	Affiliation
1	Australia	Fabienne REISEN	Commonwealth Scientific and Industrial Research Organization (CSIRO) Oceans and Atmosphere/Climate Science Centre
2	Bangladesh	Mahbuba YESMIN	Internal Medicine Department, Apollo Hospital, Dhaka
3	Bangladesh	Abdus SALAM	Department of Chemistry, University of Dhaka
4	India	Swastik BHARDWAJ	All India Institute of Medical Sciences Bhopal
5	India	Harshita PAWAR	Department of Earth and Environmental Sciences, Indian Institute of Science Education and Research, Mohali
6	Indonesia	Ir. Puji LESTARI	Faculty of Civil and Environmental Engineering, Institute of Technology Bandung
7	Indonesia	Dwi AGUSTIAN	Division of Epidemiology and Biostatistics, Dept. of Public Health, Faculty of Medicine, Universitas Padjadjaran
8	Japan	Giles Bruno SIOEN	Future Earth Global Hub—Japan
9	Japan	Hein MALLEE	Regional Centre for Future Earth in Asia; Research Institute for Humanity and Nature
10	Japan	Hiroshi TANIMOTO	International Global Atmospheric Chemistry (IGAC); National Institute for Environmental Studies
11	Japan	Tatsuya NAGASHIMA	Regional Atmospheric Modeling Section, Center for Regional Environment Research, National Institute for Environmental Studies
12	Japan	Lina MADANIYAZI	Department of Pediatric Infectious Diseases, Nagasaki University
13	Korea	Kiyoung LEE	Department of Environmental Health Sciences, Graduate School of Public Health, Seoul National University
14	Korea	Sooyoung GUAK	Environmental Health, School of Public Health, Seoul National University
15	Malaysia	Mohd Talib LATIF	School of Environmental and Natural Resource Sciences, Faculty of Science and Technology, Universiti Kebangsaan Malaysia
16	Malaysia	Mazrura SAHANI	Center for Health and Applied Sciences, Faculty of Health Sciences, Universiti Kebangsaan Malaysia
17	Malaysia	Mohd Nordin HASAN	Regional Centre for Future Earth in Asia
18	Myanmar	Ohnmar May Tin HLAING	Environmental Health Consultant Environmental Quality Management Co., Ltd.
19	Nepal	Yadav Prasad JOSHI	Environmental Health and Occupational Health, Manmohan Memorial Institute of Health Sciences
20	Pakistan	Muhammad Fahim KHOKHAR (On-Line)	Institute of Environmental Sciences and Engineering, National University of Sciences and Technology
21	Pakistan	Ejaz Ahmad KHAN (On-Line)	Health Services Academy
22	Philippines	Maria Obiminda L. CAMBALIZA	Department of Physics, Ateneo de Manila University/Air Quality Dynamics Laboratory, Manila Observatory
23	Philippines	John Q. WONG	Ateneo School of Medicine and Public Health, Ateneo de Manila University
24	Taiwan	Wen-Cheng WANG	Research Center for Environmental Change, Academia Sinica
25	Taiwan	Shih-Chun Candice LUNG	Research Center for Environmental Changes, Academia Sinica
26	Thailand	Nguyen Thi Kim OANH	Environmental Engineering and Management, Asian Institute of Technology (AIT)
27	Thailand	Kraichat TANTRAKARNAPA	Department of Social and Environmental Medicine, Faculty of Tropical Medicine, Mahidol University
28	Vietnam	To THI HIEN	University of Science, Vietnam National University, Ho Chi Minh City
29	Vietnam	Tran Ngoc DANG	Environmental Health Department, University of Medicine and Pharmacy, Ho Chi Minh City
